# Gene-environment interactions due to quantile-specific heritability of triglyceride and VLDL concentrations

**DOI:** 10.1038/s41598-020-60965-9

**Published:** 2020-03-11

**Authors:** Paul T. Williams

**Affiliations:** Lawrence Berkeley National Laboratory, Molecular Biophysics & Integrated Bioimaging Division 1 Cyclotron Road, Berkeley, CA 94720 USA

**Keywords:** Genetic association study, Genetic interaction, Genetic markers, Medical genetics, Risk factors

## Abstract

“Quantile-dependent expressivity” is a dependence of genetic effects on whether the phenotype (e.g., triglycerides) is high or low relative to its distribution in the population. Quantile-specific offspring-parent regression slopes (β_OP_) were estimated by quantile regression for 6227 offspring-parent pairs. Quantile-specific heritability (*h*^2^), estimated by 2β_OP_/(1 + r_spouse_), decreased 0.0047 ± 0.0007 (P = 2.9 × 10^−14^) for each one-percent decrement in fasting triglyceride concentrations, i.e., *h*^2^ ± SE were: 0.428 ± 0.059, 0.230 ± 0.030, 0.111 ± 0.015, 0.050 ± 0.016, and 0.033 ± 0.010 at the 90th, 75th, 50th, 25th, and 10th percentiles of the triglyceride distribution, respectively. Consistent with quantile-dependent expressivity, 11 drug studies report smaller genotype differences at lower (post-treatment) than higher (pre-treatment) triglyceride concentrations. This meant genotype-specific triglyceride changes could not move in parallel when triglycerides were decreased pharmacologically, so that subtracting pre-treatment from post-treatment triglyceride levels necessarily created a greater triglyceride decrease for the genotype with a higher pre-treatment value (purported precision-medicine genetic markers). In addition, sixty-five purported gene-environment interactions were found to be potentially attributable to triglyceride’s quantile-dependent expressivity, including gene-adiposity (*APOA5*, *APOB*, *APOE*, *GCKR*, *IRS-1*, *LPL*, *MTHFR*, *PCSK9, PNPLA3, PPARγ*2), gene-exercise (*APOA1, APOA*2*, LPL*), gene-diet (*APOA5, APOE, INSIG*2*, LPL, MYB, NXPH1, PER*2*, TNFA*), gene-alcohol (*ALDH*2*, APOA5, APOC3, CETP, LPL)*, gene-smoking (*APOC3, CYBA, LPL, USF1)*, gene-pregnancy (*LPL*), and gene-insulin resistance interactions (*APOE*, *LPL)*.

## Introduction

Obesity, physical inactivity, high-carbohydrate diets, alcohol intake, smoking, pregnancy, and type 2 diabetes mellitus (T2DM) all increase triglyceride concentrations^1^. The magnitude of the increase varies substantially across individuals, which has been attributed in part to gene-environment interactions.

An alternative to gene-environment interaction is quantile-dependent expressivity, i.e., a dependence of genetic effects upon whether the phenotype (e.g., triglycerides) is high or low relative to its distribution in the population^2^. Specifically, different genetic effects can be obtained by selecting subjects for characteristics that distinguish high vs. low portions of the triglyceride distribution. We have shown that the effect size of a 31-SNP genetic risk score (GRS_TG_) increased significantly with increasing percentile of the triglycerides distribution^2^. Specifically, the effect of the GRS_TG_ on triglyceride concentrations was 3.3-fold greater at the 90^th^ percentile of the triglyceride distribution than at its 10^th^ percentile. Within individuals, we have also shown that the genetic effect size for polymorphisms associated with *ABCA1, APOA1, APOA*2*, APOA4, APOA5, APOB, APOC3, APOE, CETP, FABP*2*, FATP6, GALNT*2*, GCKR, HL, IL1b, LEPR, LOX-1, LPL, MC4R, MTP, NPY, SORT1, TNFA, TCF7L*2, and *TM6SF*2 became significantly greater as the average triglyceride concentrations over all genotypes increased during postprandial lipemia^3^. Quantile-dependent expressivity has also been demonstrated for total cholesterol^2^, high- and low-density lipoprotein cholesterol^2^, body mass index^2^, and coffee consumption^4^.

Only about 11% of the triglyceride variance is currently explained by the 36 single nucleotide polymorphisms (SNP) showing genome-wide significance for fasting plasma triglyceride concentrations^5,6^. In contrast, heritability *(h*^*2*^) calculated from monozygotic twins raised together and apart suggest that additive genetic effects account for 54% to 65% of the triglyceride variance^7^. Verification of quantile-dependent expressivity was therefore sought using a more inclusive genetic measure in a larger population. To this end, we applied quantile regression^8,9^ to sibships and offspring-parent pairs from the Framingham Study^10,11^ to estimate heritability in the narrow sense (*h*^*2*^^12^) at different quantile of the plasma triglyceride and very-low-density lipoprotein (VLDL) cholesterol distributions. Its importance is illustrated in fourteen published reports of drug or other treatment by genotypes interactions that were originally interpreted from a precision-medicine perspective^13–25^, and sixty-five other published examples originally attributed to biological interactions between genes and environment^26–80^, which might be more simply ascribed to quantile-dependent expressivity.

## Methods

The Framingham Study data were obtained from the National Institutes of Health FRAMCOHORT, GEN3, FRAMOFFSPRING Research Materials from the NHLBI Biologic Specimen and Data Repository Information Coordinating Center. The Offspring Cohort consisted of 5,124 adult children of the original Framingham Study participants and their spouses who were first examined between 1971 and 1975, reexamined eight years later, and then every three to four years thereafter^10^. Children of the Offspring Cohort were recruited to form the Third Generation Cohort^11^. Subjects used in the current analyses were at least 16 years of age and were not taking medications to control lipid levels. Triglyceride concentrations were measured fluorometrically^81^ for all 9 exams of the Offspring Cohort, and exams 1 and 2 of the Third Generation Cohort. VLDL-cholesterol at exams 1–3 of the Offspring Cohort was determined by subtracting the bottom fraction cholesterol from total cholesterol^81^. Individual subject triglyceride values calculated as the average of the age- and sex-adjusted triglyceride concentrations over all available exams (i.e. the average of up to 9 exams for the Offspring Cohort, and up to 2 exams for Third Generation Cohort). VLDL-cholesterol was the average of up to three age- and sex-adjusted measurements.

Offspring-parent correlations (r_OP_) and regression slopes (β_OP_) were computed by assigning a weight of one-half to the offspring-father and one-half to the offspring-mother pair (if both parents were available), and assigning a weight of one to the offspring-parent pair if only one parent was available. Age and sex adjustment was performed separately in the Offspring and Third Generation Cohorts using standard least-squares regression with the following independent variables: female (0,1), age, age^2^, female x age, and female x age^2^. Offspring-midparental correlations (r_OM_) and regression slopes (β_OM_) were computed by comparing each child’s age- and sex-adjusted value to the average of the age and sex-adjusted parental values in those families having both parents. Full-sibling correlations (r_FS_) and regression slopes (β_FS_) were obtained by forming all k_i_(k_i_-1) sibpair combinations for the k_i_ siblings in sibship i and assigning equal weight to each sibling^82^. The Lawrence Berkeley National Laboratory Human Subjects Committee approved use of the Framingham Cohort data for analysis.

Simultaneous quantile regression was performed using the sqreg command of Stata (version. 11, StataCorp, College Station, TX) and one thousand bootstrap samples were drawn to estimate the variance-covariance matrix for the 91 quantile regression coefficients between the 5^th^ and 95^th^ percentiles of the offsprings’ triglyceride distribution^8,9^. Post estimation procedures (test and lincom) were used to test linear combinations of the slopes with Σk_i_ -2 degrees of freedom for β_OP_ and Σ (k_i_-1) degrees of freedom for β_FS_, where k_i_ is the number of offspring in family i, and the summation is taken over all family sets. Quantile-specific expressivity was assessed by: 1) estimating quantile-specific β_OP_ ± SE and β_FS_ ± SE for the 5^th^, 6^th^… 95^th^ percentiles of the sample distribution using simultaneous quantile regression; 2) plotting the quantile-specific β_OP_ and β_FS_ coefficients vs. the quantile of the offsprings’ trait distribution; and 3) testing whether the resulting graphs was constant, or changed as a linear, quadratic, or cubic function of the percentile of the trait distribution using orthogonal polynomials. Heritability in the narrow sense (*h*^*2*^) was estimated as *h*^*2*^ = 2β_OP_/(1 + r_spouse_) from the offspring-parent regression slope (β_OP_), as *h*^*2*^ = β_OM_ from the offspring-midparental regression slope (β_OM_), and as *h*^*2*^ = [(1 + 8r_spouse_β_FS_)^0.5^−1]/(2r_spouse)_ from full-sibs regression slopes (β_FS_), where r_spouse_ is the spouse correlation^12^.

Published reports of gene-treatment and gene-environment interactions were identified through PubMed and the citations within each paper retrieved. In many cases^27–30,32,33,35,36,40,43,48,49,62,64,65,67,70^, mean triglyceride concentrations had to be estimated from published figures using the formatting palette of Microsoft Powerpoint to extract their quantitative information (version 12.3.6 for Macintosh computers, Microsoft corporation, Redmond WA). Vertical lines were drawn showing the vertical distances between each plotted point and the X-axis, and overall height of the Y-axis, from which triglyceride concentrations were derived^3^.

## Results

There were 3325 Third Generation subjects who had one or more parents in the Offspring Cohort (1089 had one parent, 2236 had both parents). There were 1016 sibships with two or more full siblings in the Offspring Cohort (532 with two, 302 with three, 122 with four, and 60 with ≥five full sibs) and 1171 sibships with two or more full siblings in the Third Generation Cohort (576 with two, 333 with three, 155 with four, and 107 with ≥five full sibs). Unadjusted average triglyceride (SD) for subjects used in the analyses was 2.390 (1.934) mmol/L in the Offspring Cohort and 1.279 (0.914) mmol/L in the Third Generation Cohort. In addition, sibships from the Offspring Cohort had an unadjusted average VLDL-cholesterol concentration of 0.585 (0.294) mmol/L for exams 1–3.

Correlational analyses showed spouses were concordantly related for age- and sex-adjusted triglycerides (r_spouse_ = 0.15), log triglycerides (r_spouse_ = 0.31), and VLDL-cholesterol (r_spouse_ = 0.09). Table 1 presents the traditional least squares regression slopes between offspring and parent (β_OP_) and offspring and midparent (β_OM_) and among full sibs (β_FS_). Triglyceride heritability (*h*^*2*^ ± SE) was significant as traditionally estimated from β_OP_ (0.146 ± 0.013), β_OM_ (0.131 ± 0.012), or β_FS_ (0.456 ± 0.031). Heritability was even stronger for log triglycerides when estimated from β_OP_ (0.360 ± 0.023), β_OM_ (0.380 ± 0.023), or β_FS_ (0.532 ± 0.030). Heritibility of VLDL-cholesterol was 0.343 ± 0.046 when estimated from full sibs (β_OP_ unavailable because parents were not measured).

**Table 1:** Traditional and quantile regression analyses of triglycerides and very low density lipoprotein (VLDL)-cholesterol from the Offspring and Third Generation Framingham Cohorts.

	Least-squares regression analysis			Quantile regression analysis					
		Traditional regression slope		Increase in slope per 1% increase in the percentile of the dependent variable’s distribution				Difference in slope between the 90^th^ and 10^th^ percentiles	
				Linear effect		Nonlinear effects			
	Correl-ation	Slope ± SE	Sig (P)	Slope ± SE	Linear (P)	Quadratic (P)	Cubic (P)	Difference ± SE	Sig (P)
**Offspring Parent-**									
Triglycerides	0.18	0.0837 ± 0.0074	10^−15^	0.0027 ± 0.0004	2.9 × 10^−14^	1.7 × 10^−6^	0.0007	0.2269 ± 0.0339	2.2 × 10^−11^
Log triglycerides	0.25	0.2357 ± 0.0152	10^−15^	0.0023 ± 0.0004	4.9 × 10^−10^	0.65	0.11	0.1902 ± 0.0391	1.1 × 10^−6^
**Offspring-Midparent**									
Triglycerides	0.22	0.1311 ± 0.0117	10^−15^	0.0035 ± 0.0006	6.5 × 10^−9^	0.004	0.08	0.2751 ± 0.0763	0.0003
Log triglycerides	0.32	0.3801 ± 0.0227	10^−15^	0.0030 ± 0.0007	1.3 × 10^−5^	0.46	0.65	0.2218 ± 0.0787	0.005
**Full Sibling**									
Triglycerides	0.24	0.2434 ± 0.0154	10^−15^	0.0042 ± 0.0007	1.8 × 10^−9^	0.0007	0.003	0.3662 ± 0.0791	3.6 × 10^−6^
Log triglycerides	0.31	0.3096 ± 0.0151	10^−15^	0.0011 ± 0.0004	0.007	0.05	0.68	0.0747 ± 0.0360	0.04
VLDL-cholesterol	0.18	0.1767 ± 0.0231	2.2 × 10^−14^	0.0026 ± 0.0009	0.003	0.12	0.24	0.1974 ± 0.0945	0.04

Triglycerides and log triglycerides: 1174 offspring with one parent and 2507 with two parents, and 6176 full siblings in 2187 sibships; VLDL-cholesterol: 2840 full siblings in 1029 sibships.

### Plasma triglyceride concentrations

**Figure 1:**
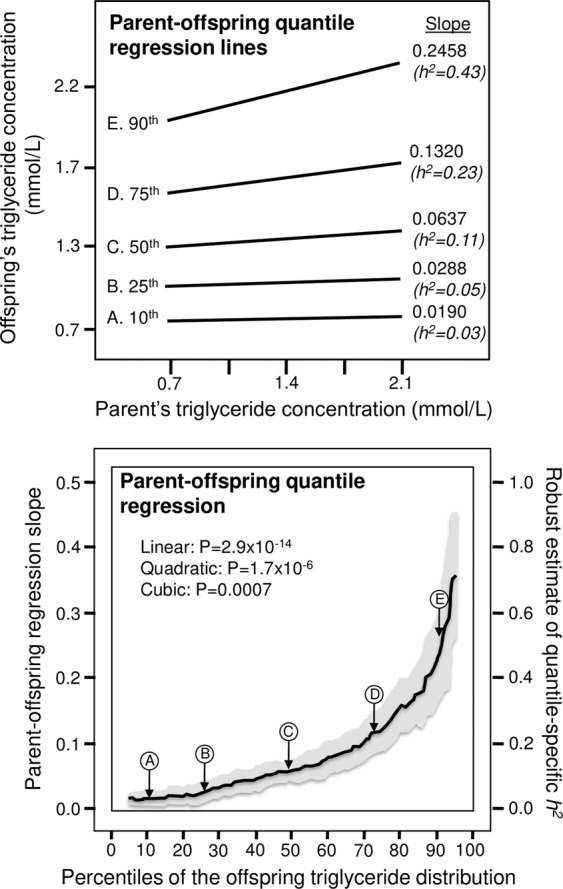
(upper panel) presents the offspring-parent regression slopes (β_OP_) for selected quantiles of the offsprings’ total triglyceride concentrations, with corresponding estimates of heritability (*h*^*2*^ = 2β_OP_/(1 + r_spouse_)). The slopes became progressively greater (i.e., steeper) with increasing quantiles of the triglyceride distribution. These quantile-specific regression slopes were included with those of other quantiles to create the quantile-specific heritability function in the lower panel. The statistical significance of the linear, quadratic and cubic trends and the 95% confidence intervals (shaded region) were determined by 1000 bootstrap samples. 1 mg/dL = 0.01129 mmol/L.

Figure 1 (upper panel) presents the offspring-parent regression slopes (β_OP_) for selected quantiles of the offspring’s plasma triglyceride distribution with their associated heritability estimates (*h*^*2*^ the narrow sense). Heritability became progressively greater with increasing quantiles of the offspring’s distribution, and differed significantly between the 10^th^ and 90^th^ percentiles (P =  2.2 × 10^−11^). These selected quantile-specific heritability estimates were included with those of other quantiles to create the quantile-specific heritability function in the lower panel, i.e., where *h*^*2*^ (Y-axis) is plotted as a function of the quantile of the offspring’s sample distribution (X-axis). Specifically, the Y-axis represents heritability at the 5th quantile, the 6th quantile,…, and the 95th quantiles of the offspring’s distribution. The shaded area presents the 95% confidence intervals for the individual slopes at each quantile. The figure shows that *h*^2^ increased from 0.033 ± 0.010 at their 10th percentile (P = 0.0009), 0.050 ± 0.016 at the 25th (P = 0.001), 0.111 ± 0.015 at the 50^th^ (P = 1.3 × 10^−13^), 0.230 ± 0.030 at the 75^th^ (P = 1.7 × 10^−14^), and 0.428 ± 0.059 at the 90th percentile of the offspring’ distribution (P = 6.4 × 10^−13^). If the heritability was the same for all offspring quantiles as traditionally assumed, then the upper panel would display parallel regression lines, and the lower graph would present a simple horizontal line. In fact, the graph shows that heritability became progressively stronger with increasing quantiles of its offsprings’ triglyceride distribution, such that on average each 1-percent increase in the offspring distribution was associated with a 0.0047 ± 0.0007 increase in heritability (P = 2.9 × 10^−14^). Moreover, the increase in quantile-specific *h*^2^ with increasing offspring’s triglyceride concentrations was significantly nonlinear, exhibiting both quadratic (P = 1.7 × 10^−6^) and cubic (P = 0.0007) effects. With respect to individual quantiles, heritability was statistically significant (P < 0.003) at every percentile between the 5^th^ and the 95^th^ percentiles of the offspring’ distribution, and was 13-fold greater at the 90th than at the 10th percentile.

### Full-sib quantile regression

**Figure 2:**
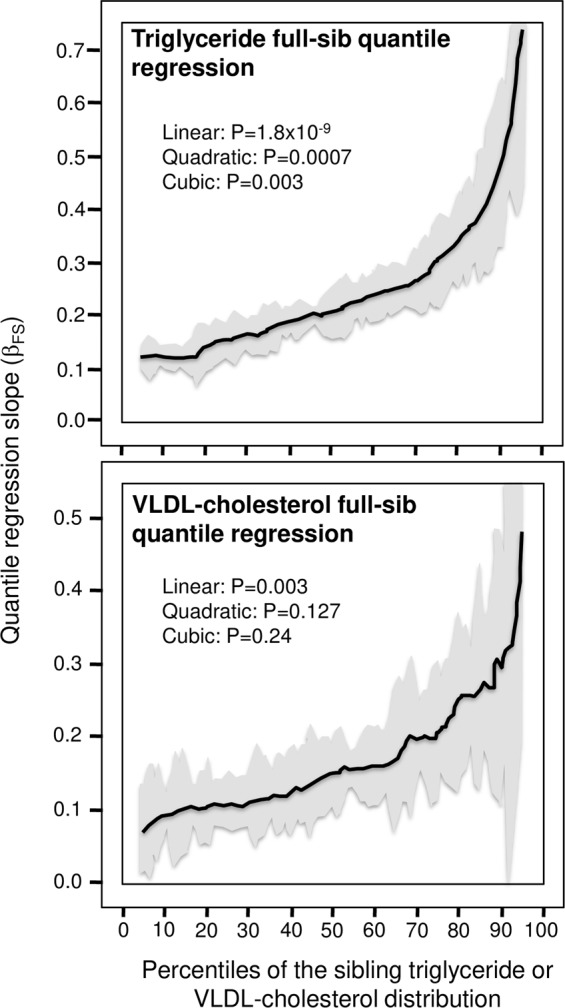
Full-sib regression slopes (β_FS_) vs. quantiles of the sib’s triglyceride and VLDL-cholesterol distribution.

Figure 2 shows that the full-sib regression slope for triglyceride concentrations (β_FS_): 1) was 3.8-fold greater at the 90^th^ (0.487 ± 0.081) than the 10^th^ percentile (0.121 ± 0.010) of the sib distribution; and 2) increased 0.0042 ± 0.0007 (P = 1.8 × 10^−9^) for each percentile increase in the sibs’ distribution, and 3) exhibited significant nonlinearity (quadratic: P = 0.0007; cubic: P = 0.003). The full-sib slopes were statistically significant (P < 0.0001) at every percentile between the 5^th^ and the 95^th^ percentiles of the offspring’ distribution. Figure 2 also shows that siblings exhibited quantile-specific associations that were significantly greater at the 90^th^ than 10^th^ percentiles of the VLDL-cholesterol distribution (P = 0.04), and exhibited significant linear increases with each one-percent increment in their VLDL-cholesterol (0.0026 ± 0.0009, P = 0.003).

### Log-transformed triglyceride concentrations

**Figure 3:**
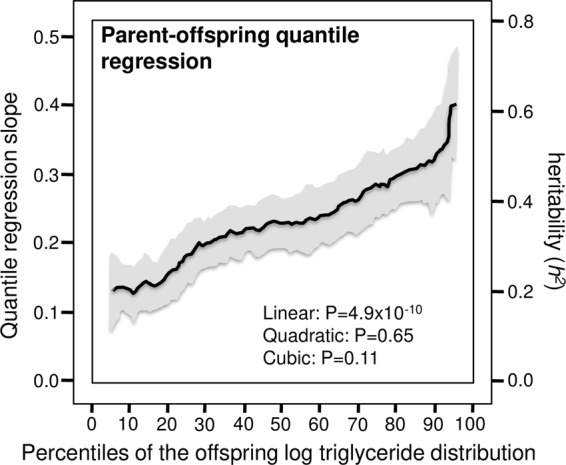
Offspring-parent regression slopes (β_OP_) vs. quantiles of the offsprings’ log-transformed triglyceride concentrations.

Quantile-dependent effects persisted when triglyceride concentrations were log transformed for offspring-parent (Fig. 3, P = 4.9 × 10^−10^) and full-sib regression slopes (Table 1, P = 0.007).

### Replication

Significant quantile-specific increases in β_FS_ were detected separately for fasting triglycerides measured in 2792 sibling in 1016 sibships in the Framingham Offspring Cohort (linear: P = 7.7 × 10^−5^; quadratic: P = 0.003; cubic: P = 0.003), and in 3384 sibling in 1171 sibships in the Framingham Third Generation Cohort (linear: P = 0.001; quadratic: P = 0.84; cubic: P = 0.55).

## Discussion

Genome-wide association studies have identified 36 single nucleotide polymorphisms (SNP) associated with triglyceride concentrations^5,6^. The most significant SNPs are associated with the glucokinase regulator (*GCKR*, P = 2 × 10^−239^), apolipoprotein A1 *(APOA1*, P = 7 × 10^−224^), and lipoprotein lipase genes (*LPL*, P = 2 × 10^−199^). Because only about 11% of the triglyceride variance is explained by these 36 loci^5,6^, the current paper investigated heritability in the narrow sense (*h*^*2*^) as a more comprehensive, albeit less specific, estimate of genetic transmission. It showed that *h*^2^ increased significantly with increasing percentiles of the triglyceride distribution. This result was replicated for β_FS_ in the Framingham Offspring Cohort and Framingham Third Generation cohort separately. This confirms our previous analyses of fasting plasma triglyceride concentrations vs. GRS_TG_^2^, and postprandial triglyceride concentrations vs. individual SNPs^3^. The current analyses also demonstrated quantile-dependency for VLDL-cholesterol concentrations in sibs. Quantile dependence was also significant for log-transformed triglyceride concentrations. These analyses were based on simple robust estimates of heritability with nonparametric statistical significance determined from 1000 bootstrap samples.

### Pharmacogenetics

Quantile-dependent expressivity predicts that genes affecting triglyceride concentrations should have a greater genetic effect prior to drug treatment when concentrations are high, than post-treatment when triglycerides are low. Moreover, smaller genotype differences when triglycerides concentrations are reduced pharmacologically might appear as gene-drug interactions in the absence of any true biological interactions. This prediction was assessed in 14 published reports purporting gene-drug or gene-treatment interactions on triglyceride response^13–25^.

**Figure 4:**
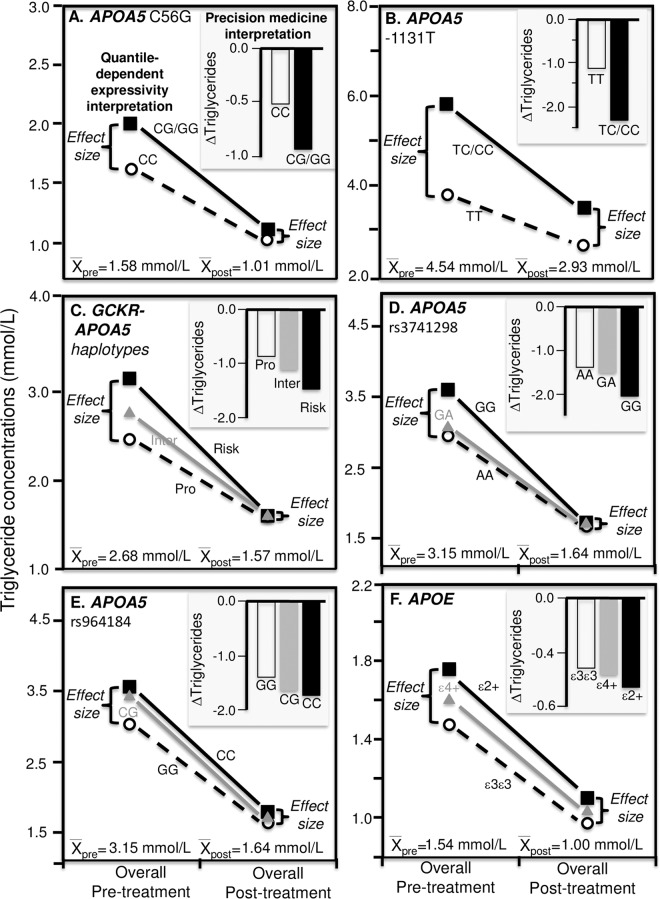
Precision medicine perspective of different mean triglyceride reductions by genotypes following 160 mg/d fenofibrate or fenofibrate/statin combination therapy (histogram inserts of mean changes by genotype) vs. quantile-dependent expressivity interpretation (larger pre-treatment genetic effect size when average triglycerides concentrations were high vs. lower, requiring nonparallel triglycerides reductions by genotype), for: (**A**) Lai *et al*.’s 2007 report of 87 *APOA5* 56 G carriers vs. 703 non-carriers (genotype difference in mean triglyceride reduction P = 0.006)^15^; (**B**) Cardona’s *et al*.’s 2009 report of 14 *APOA5* -1131C carriers vs. 22 non-carriers^17^; (**C**) Perez-Martinez *et al*.’s 2009 report of *protected group* (N = 236) consisting of the common allele homozygotes for *GCKR* rs780094C > T (CC), *APOA5* −1131 T > C (TT), and *APOA5* 56 C > G (CC); an *intermediate group* (N = 490) consisting of homozygotes for *GCKR* rs780094C > T (CC) and carriers of the rare allele for either *APOA5* −1131 T > C (CT or CC) or *APOA5* 56 C > G (CG or GG) or carriers of the rare allele for *GCKR* rs780094C > T (CT or TT) and homozygotes for both *APOA5* −1131 T > C (TT) and *APOA5* 56 C > G (CC); and a *risk group* (N = 118) consisting of carriers of the rare allele for *GCKR* rs780094C > T (CT or TT) and carriers of the rare allele for either *APOA5* −1131 T > C (CT or CC) or *APOA5* 56 C > G (CG or GG) with triglycerides > 1.69 mmol/L at baseline^16^; (**D**) Brautbar *et al*.’s 2011 report on 47 GG, 256 GA and 371 AA genotypes of rs3741298 in the *APOA5-ZNF259* gene region who were also statin treated^14^; (**E**) Brautbar *et al*.’s 2011 report on 27 CC, 202 CG and 445 GG genotypes of rs964184 in the *APOA5-ZNF259* gene region who were also statin treated^14^; and (**F**) Irvin *et al*.’s 2010 report on 81 *APOE* ε2-carrier, 454 ε3ε3, and 203 ε4-carriers^18^.

For example, the histogram in Fig. 4A (insert) shows the reductions in fasting triglyceride levels reported by Lai *et al*. after three-week fenofibrate treatments^15^. The average decrease was significantly greater in *APOA5* 56 G carriers than non-carriers (35.8% vs. 27.9% decreases, P = 0.006). An accompanying editorial heralded its potential contribution to personalized medicine^83^. There is, however, an alternative interpretation of Lai *et al*.’s results from the perspective of quantile-dependent expressivity. Figure 4A shows that average triglyceride levels were higher before (1.58 ± 0.04 mmol/L) than after treatment (1.01 ± 0.02 mmol/L) and that triglyceride difference between genotypes were greater at the higher pre-treatment triglyceride levels (1.99–1.52 = 0.46 mmol/L difference, P = 0.01) than at the lower post-treatment triglyceride levels (1.06–1.00 = 0.06 mmol/L difference, P = 0.22), consistent with quantile-dependent expressivity. The smaller genetic effect size at the lower (post-treatment) than higher (pre-treatment) average triglyceride concentration requires that the trajectories of triglyceride reductions cannot move in parallel for different genotypes when triglycerides are decreased pharmacologically. Subtracting the pre-treatment from the post-treatment triglyceride levels will necessarily require a relatively greater triglyceride decrease for the genotype with the higher pre-treatment triglyceride level vis-à-vis the genotype with the lower pre-treatment level.

**Figure 5:**
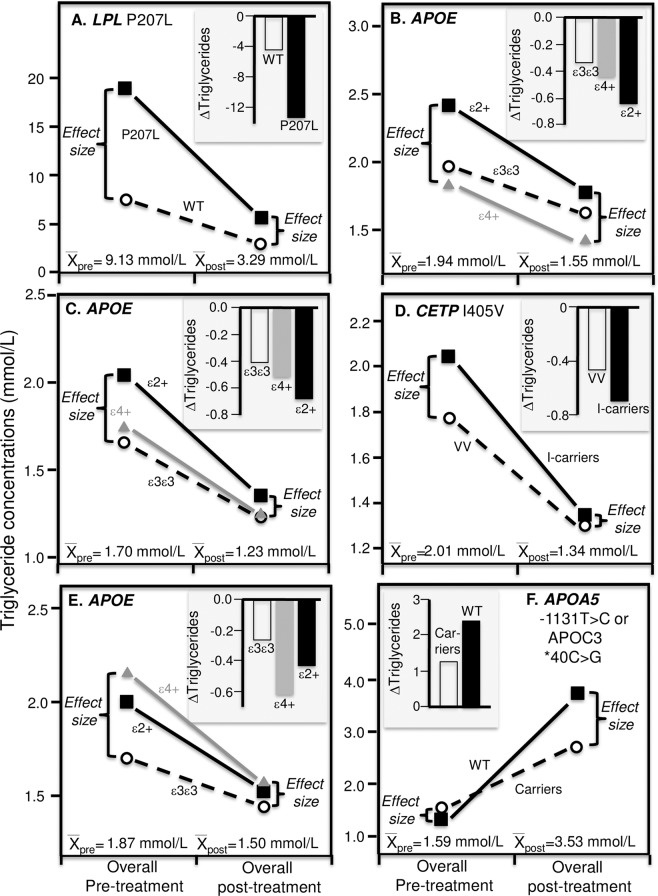
Precision medicine perspective of mean changes in triglyceride concentrations by genotypes (histogram inserts) vs. quantile-dependent expressivity perspective of larger genetic effect size when average triglycerides concentrations were high vs. low requiring nonparallel changes in triglycerides by genotype, for: (**A**) Brisson *et al*.’s 2015 report on 160 mg/d fenofibrate therapy in 44 carriers of *LPL* P207L mutation vs. 247 non-mutants^19^; (**B**) Pedro-Botet *et al*.’s report on 10 mg/day of atorvastatin’s effect in 10 male *APOE* ε2-carriers, 111 male ε3ε3, and 74 male ε4-carriers^20^; (**C**) Carmena *et al*.’s 2012 report on 80 mg/d lovastatin’s effect on 7 *APOE* ε2+, 58 ε3ε3, and 29 ε4+ familial hypercholesterolemia (FH) patients^21^; (**D**) Anagnostopoulou *et al*. 2007 report of 10–40 mg/d simvastatin in 160 carriers of the I-allele and 20 VV homozygotes of the *CEPT* I405V polymorphism^22^; (**E**) Balakrishnan *et al*.’s 2002 report of 93 patients who received pancreas transplants by *APOE* isoforms^24^; (**F**) Cabello *et al*.’s 2018 report on the effect of bexarotene treatment on carriers of the *APOA5* -1131T > C or *APOC3* 388 T > C mutations vs. non-mutations^25^.

Figures 4 and 5 display additional reports, initially interpreted from the perspective of personalized medicine, that are consistent with quantile-dependent expressivity, i.e., larger pre-treatment genetic effects when average triglycerides are high, followed by smaller post-treatment genetic effects when average triglyceride concentrations are low. Cardona *et al*. reported that the triglyceride reduction from fenofibrate treatment was over twice as great in TC/CC genotypes than TT homozygotes of the *APOA5* -1131T polymorphism (2.34 vs. 1.15 mmol/L decreases, Fig. 4B histogram)^17^. The graph show that there was a greater triglyceride difference between carriers of the C-allele and TT homozygotes before treatment (5.80–3.74 = 2.06 mmol/L) when average triglycerides were high (4.54 mmol/L) than after treatment (3.46–2.60 = 0.86 mmol/L) when average triglycerides were lower (2.93 mmol/L). Perez-Martinez *et al*^16^. identified three genetic risk groups in hypertriglyceridemic subjects (pre-treatment triglycerides >1.69 mmol/L) derived from the *GCKR*-*APOA5* loci: a *protected group*, an *intermediate group*, and a *risk group*. The histogram in Fig. 4C shows the decreases in plasma triglyceride concentration differed significantly between these groups after three-week fenofibrate treatment (P = 0.003): greatest in the *risk group*, intermediate in the *intermediate group*, and least in the *protected group*. However, the cross-sectional genotype differences were greater at baseline (risk: 3.08; intermediate: 2.71; protected: 2.40 mmol/L, P = 0.009) when the average triglyceride concentration over all genotypes was high (2.68 mmol/L), than after treatment (all genotypes approximately the same, P = 0.20) when average triglycerides were low (1.57 mmol/L, estimated from their Fig. 3^16^).

Brautbar *et al*. reported that three SNPs in the *ZNF*2*59-APOA5* gene region on chromosome 11 showed substantially smaller genotype differences on fenofibrate/statin combination treatment when average triglyceride levels were low (1.64 mmol/L) compared to pretreatment differences when average levels were higher (3.15 mmol/L)^14^. Specifically, treatment reduced differences between GG, GA, and AA genotypes of rs3741298 from 3.77, 3.20, and 3.04 (P = 3.2 × 10^−5^) to 1.67, 1.65, and 1.64, respectively (P = 0.79, Fig. 4D), between the CC, CG and GG genotypes of rs964184 from 3.51, 3.41, 3.01 mmol/L (P = 2.3 × 10^−7^) to 1.77, 1.71, and 1.61 mmol/L, respectively (P = 0.18, Fig. 4E), and between GG, GA, and AA genotypes of rs10750097 from 3.37, 3.26, and 3.05 mmol/L (P = 0.002) to 1.62, 1.66, and 1.63 mmol/L, respectively (P = 0.86). Although the mean triglyceride reductions by genotype did not differ significantly by genotype in Brautbar’s paper (0.25 ≤ P ≤ 0.82), one by Aslibekyan *et al*. did report that the rs964184 polymorphism affected fenofibrate-induced triglyceride change significantly (P < 0.001)^13^.

Irvin *et al*.^18^ (Fig. 4F) reported that fenofibrate-induced changes in plasma triglyceride concentrations differed significantly (P = 0.05) between *APOE* ε3ε3 homozygotes (−29.0%), ε4-carriers (−26.4%) and ε2-carriers (−34.4%), an expected result from quantile-dependent expressivity given that the triglyceride difference between ε3ε3, ε4-carriers, and ε2-carriers were greater before treatment (1.48, 1.60, 1.75 mmol/L, respectively, P = 0.02) when average triglycerides were high (1.54 ± 0.03 mmol/L) than after treatment (0.97, 1.04, 1.10 mmol/L, P = 0.09) when average triglycerides were low (1.00 ± 0.02 mmol/L).

The histogram in Fig. 5A shows substantially greater triglyceride reductions due to fenofibrate treatment in 44 carriers of *LPL* P207L mutation than in 247 non-carriers who were hypertriglyceridemic (13.3 vs. 4.5 mmol/L average reductions). Brisson *et al*. attributed the difference to the mutation’s modulating effect on the fenofibrate response^19^. Alternatively, quantile-dependent expressivity would attribute the difference to the greater genetic effect size of the mutation (18.93–7.38 = 11.5 mmol/L difference) when average triglycerides were elevated before treatment (9.13 mmol/L) compared to post-treatment genetic effect size (5.60–2.88 = 2.72 mmol/L difference) when average triglycerides were much lower (3.29 mmol/L).

Several studies report statin-induced triglyceride reductions that were genotype specific. Pedro-Botet *et al*. reported that the average decrease in plasma triglyceride concentrations was significantly affected by *APOE* isoforms in a multicentric, double-blind clinical trial of 328 patients who received 10 mg/day of atorvastatin for one year^20^. The histogram in Fig. 5B shows that ε2-carriers had the greatest average decrease (0.64 mmol/L), ε4-carriers intermediate decrease (0.44 mmol/L), and ε3ε3 the smallest average decrease (0.34 mmol/L). However, the genetic effect size of the ε2-allele vis-à-vis ε3ε3 homozygotes was greater at baseline (0.46 ± 0.26 mmol/L) when average triglycerides were higher (1.94 mmol/L) than after one-year (0.16 ± 0.19 mmol/L) when average triglycerides concentrations were reduced (1.55 mmol/L), consistent with quantile-dependent expressivity.

These results agree with an earlier report by Carmena *et al*. of 94 patients with familial hypercholesterolemia (FH) who received 80 mg lovastatin for 80 days^21^. Figure 5C’s histogram shows that ε2-carriers showed the greatest average decrease (−0.69 mmol/L), ε4-carriers intermediate decrease (−0.52 mmol/L), and ε3ε3 the smallest average decrease (−0.41 mmol/L). Again, the genetic effect size of the ε2-allele vis-à-vis ε3ε3 homozygotes was greater at baseline (0.39 ± 0.29 mmol/L difference) when average triglycerides were higher (1.70 ± 0.02 mmol/L) than after one-year of lovastatin treatment (0.12 ± 0.15 mmol/L difference) when average triglycerides concentrations were reduced (1.23 ± 0.01 mmol/L), consistent with quantile-dependent expressivity.

Anagnostopoulou *et al*. reported significantly greater triglyceride reductions in carriers of the I-allele of the *CEPT* I405V polymorphism than VV homozygotes (0.70 vs. 0.47 mmol/L decreases, P = 0.04) from 10–40 mg/day simvastatin (Fig. 5D)^22^. Average triglyceride concentrations decreased from 2.01 to 1.34 mmol/L, and accordingly the genotype difference decreased from 0.27 mmol/L before treatment to 0.05 mmol/L after.

As a final example involving lipid-lowering drugs, Tuteja *et al*. reported that the C-allele of *PDXDC1* rs3198697 accentuated the decrease in triglycerides from niacin-statin combination therapy (P = 0.02, not displayed)^23^. Once again, a larger pre-treatment genetic effect size per dose of the C-allele (0.14 mmol/L per copy) occurred when average triglycerides were high (1.88 mmol/L), and smaller post-treatment effect size occurred (β = −0.04 mmol/L per copy) when average triglycerides were low (1.35 mmol/L).

A non-pharmacological example is provided by Balakrishnan *et al*. who reported that triglyceride concentrations were significantly reduced, from 1.87 to 1.50 mmol/L, in 93 patients who received pancreas transplants (P = 0.002)^24^. The triglyceride difference between *APOE* ε4-carriers and ε3ε3 homozygotes went from being significant (0.45 mmol/L as estimated from their Fig. 2, P = 0.04) to nonsignificant (0.08 mmol/L) after the transplant, consistent with quantile-dependent expressivity (Fig. 5E).

Hypertriglyceridemia is the most common reason for discontinuing bexarotene, a drug used for treating cutaneous T-cell lymphomas^84^. Cabello *et al*. proposed that carriers of the *APOA5* -1131T > C or *APOC3* c.40 C > G mutations were the best candidates for bexarotene treatment because of their smaller triglyceride response^25^. Figure 5F presents the triglyceride differences between genotypes before and after oral bexarotene therapy while receiving prophylactic hypolipidemic therapy and 50 μg/d of levothyroxine sodium. From the perspective of personalized medicine, carriers of either minor allele experienced smaller triglyceride increases than non-carriers (1.25 vs. 2.39 mmol/L), whereas quantile dependent-expressivity would ascribe some of the effect to the smaller genetic difference between carriers and non-carriers before treatment (effect size: + 0.12 mmol/L) when average triglyceride concentrations were lower (1.59 mmol/L) than after treatment (effect size: −1.01 mmol/L, P = 0.02) when average triglyceride concentrations were higher (3.53 mmol/L).

To summarize, whereas other papers advocate individualized drug prescriptions using genetic markers to target patients (e.g., the histograms of Figs. 4 and 5), quantile-dependent expressivity postulates that these genetic markers follow different trajectories due to smaller genetic effects at lower triglyceride concentrations. It is unnecessary to hypothesize pharmacologic interactions of these genetic markers with treatment, rather *APOA5, GCKR*, *APOA1*, and *APOE* are simply among the brightest genetic signals tracking the reduced heritability.

### Implications regarding gene-environment interactions

Environmental factors that distinguish higher vs. lower triglycerides (e.g., obesity, physical inactivity, smoking, alcohol, high-carbohydrate diets, T2DM) are predicted to produce different genetic estimates under quantile-dependent expressivity. Traditionally, these differences have been attributed to gene-environment interactions, where: 1) the effect of the genotype on the phenotype differs by environment^26–37,41–43,45–55,58–63,65–67,74–80^, or equivalently: 2) the effect of the environment on the phenotype differs by genotype^36,38–40,44,56,57,59,64,68–72^. In almost every case, these were explicitly interpreted as arising from a biological interaction between gene product and treatment. Not one of the cited reports considered the differences in average triglyceride levels between environmental conditions as an explanation of their observed results. As a causal model, quantile-dependent expressivity may arise from concentration-dependent effects of the mutations affecting triglyceride concentrations, e.g., impaired catabolism due to slower lipoprotein lipase activity having a greater effect when triglycerides were high than when low. Biologically, and from the perspective of chemical reactions, this makes more sense than the traditional fixed effect size. In our heritability analyses, it is possible that shared environmental effects contributed to offspring parent-regression slopes and that these too were quantile dependent. In contrast, our original GRS_TG_^2^ and the published examples to follow^26–79^, show quantile-dependent expressivity for genetic variants that are independent of shared environmental effects. The examples to follow represent interactions that are consistent with quantile-dependent expressivity because they show larger genetic effect sizes at higher average triglyceride concentrations.

### Body mass index and waist circumference

Meta-analyses suggest that plasma triglyceride concentrations decrease 0.015 mmol/L per kg of weight loss^85^. BMI and waist circumference are associated with higher triglyceride concentrations due, at least in part, to the release of free fatty acids from visceral depots causing greater hepatic synthesis of VLDL^86^. Most reports of gene-weight interactions appear to be at least partially attributable to quantile-dependent expressivity, including six studies based on genetic risk scores (GRS_TG_)^26–31^. Cole *et al*. reported that the effect of their GRS_TG_ on triglyceride concentrations was significantly larger in obese subjects (effect size ± SE: 0.480 ± 0.053 mmol/L for BMI ≥ 35 kg/m^2^) then in lean subjects (0.261 ± 0.034 mmol/L for BMI ≤ 23 kg/m^2^), and intermediate in subjects with an intermediate BMI (0.354 ± 0.029 mmol/L)^26^. Ali *et al*. reported that each unit increment in their GRS_TG_ was associated with a 2.4% triglyceride-increase in overweight/obese subjects, and a 1.5% triglyceride-increase in normal weight subjects^27^. Quantile-dependent expressivity would attribute these differences to the higher average triglyceride concentrations of the obese subjects reported by Cole *et al*. (>0.50 mmol/L higher) and the overweight/obese subjects reported by Ali *et al*. (estimated >0.40 mmol/L) than their lean comparison group.

Klimentidis *et al*.^28^. report that increasing tertiles of waist-to-hip ratio were associated with progressive increases in the GRS_TG_ effect size (estimated β_1st tertile_ = 0.16, β_2nd_ = 0.18, and β_3rd_ = 0.22, P_Interaction_ = 3.9 × 10^−8^). This, however, was in the context of highly significant increases in average triglyceride concentrations with increasing waist circumference (P = 1.3 × 10^−56^). Zubair *et al*. reported that the triglyceride difference between a high and low GRS_TG_ score was greater in overweight/obese women than leaner women (0.49 vs. 0.29 mmol/L, P_interaction_ = 0.03) and greater in broad-waisted than slim-waisted women (0.54 vs. 0.27 mmol/L, P_interaction_ = 0.02)^29^. Again, these differences are consistent with quantile-dependent expressivity given that average triglycerides concentrations were greater in overweight/obese than leaner women (1.38 vs. 1.22 mmol/L) and greater in broad-waisted than slim-waisted women (1.44 vs. 1.19 mmol/L, calculated from their published data).

Justesen *et al*. reported significant interactions between adiposity and their 39-SNP GRS_TG_ in two Danish cohorts: the Inter99 cohort (N = 5961 subjects) and the Health2006 cohort (N = 2565 subjects)^30^. BMI was divided into normal weight, overweight, and obese. Waist circumferences were divided into normal, centrally overweight, and centrally obese. Both cohorts showed triglycerides that were significantly affected by BMI x GRS_TG_ interactions (Inter99: P = 0.002; Health 2006: P = 0.02; combined; P = 9.8 × 10^−5^) and waist circumference x GRS_TG_ interactions (Inter99: P = 0.0001; Health 2006: P = 0.05; combined; P = 2.0 × 10^−5^), with a larger genetic effect among individuals who were obese. However, average triglyceride levels for normal weight, overweight, and obese increased from 0.92, to 1.23 to 1.55 mmol/L in the Inter99 cohort, respectively, and from 0.94 to 1.23 to 1.54 mmol/L in the Health2006 cohort. Similarly, average triglyceride levels for normal, centrally overweight, and centrally obese subjects increased from 0.96 to 1.26 to 1.49 mmol/L in the Inter99 cohort and from 0.95 to 1.18 to 1.41 mmol/L in the Health2006 cohort. From the perspective of quantile-dependent expressivity, greater adiposity was an indicator of higher average triglyceride concentrations and its larger genetic effect.

Ahmad *et al*. reported that each unit increase in their 40-SNP GRS_TG_ produced a significantly stronger effect on triglycerides in overweight and obese (1.013% triglyceride increase) than healthy weight women (1.011%, P_interaction_ = 0.004), and a significantly stronger effect in centrally overweight and obese (1.012%) than centrally healthy weight women (1.010%, P_interaction_ = 0.005)^31^. These results are consistent with quantile-dependent expressivity and the higher triglyceride concentrations of the overweight and obese vs. healthy weight women (1.8 vs. 1.3 mmol/L, P < 0.0001), and the centrally overweight and obese vs. centrally normal weight women (1.7 vs. 1.2 mmol/L, P < 0.0001).

Gene-weight interactions have also been reported for individual SNPs, including those associated with *APOA5*^26,32–34^, *LPL*^26,35–43^, *GCKR*^26,44^, insulin receptor substrate-1 (*IRS-1*)^45,46^, methylenetetrahydrofolate reductase (*MTHFR*)^47,48^, proprotein convertase subtilisin/kexin type 9 (*PCSK9*)^48^, *APOB*^49^, *APOE*^50^, peroxisome proliferator-activated receptor γ 2 (*PPARγ*2)^51^, and patatin-like phospholipase domain-containing protein 3 gene (*PNPLA3*)^52^.

The *APOA5* gene is the strongest genetic determinant of plasma triglyceride concentrations^87^. Four studies report interactions between BMI and *APOA5* polymorphisms that are consistent with quantile-dependent expressivity. Wu *et al*. reported that the effect size for the Gly185Cys polymorphism at *APOA5* rs3741297 was accentuated in Filipinos with a higher waist circumference^32^. Specifically, it increased from 0.13, 0.06, 0.30, to 0.96 mmol/L from the first to the fourth quartiles of waist circumference in mothers (P_interaction_ = 0.01), and from 0.19, 0.03, 0.13, to 0.58 mmol/L from the first to the fourth quartiles in offspring (P_interaction_ = 0.007). Average triglycerides levels also increased from the first to the fourth quartiles of waist circumference, i.e., 0.96, 1.24, 1.38, 1.55 mmol/L in mothers, and 0.86, 0.90, 1.03, and 1.23 mmol/L in offspring. A second study, by Kim *et al.*^33^, reported that the triglyceride difference between C-carriers and TT homozygotes of the *APOA5* -1131T > C polymorphism was greater in overweight vs. normal weight Koreans at baseline (0.31 vs. 0.10 mmol/L) and their 3-year follow-up (0.55 vs. 0.19 mmol/L), corresponding to the higher average triglycerides in overweight than normal weight Koreans at baseline (1.47 vs. 1.02 mmol/L) and follow-up (1.60 vs. 1.06 mmol/L, estimated from their published graphs). Hsu *et al*.’s reported that the effect of the C-allele of rs662799 on plasma triglyceride concentrations was greater in obese than lean patients (0.473 vs. 0.142 mmol/L per C-allele) in accordance with their higher average triglyceride concentrations (1.51 ± 0.07 vs. 0.90 ± 0.02 mmol/L)^34^. The fourth study, by Cole *et al*.^26^., reported a significantly greater effect size for *APOA5* rs964184 in obese than lean subjects (β = 0.159 ± 0.03 vs. 0.140 ± 0.03 mmol/L per G allele, P_interaction_ = 0.009) whose average triglycerides differed by >0.5 mmol/L.

The LPL enzyme hydrolyzes triglycerides, and it participates in hepatic triglyceride-rich lipoprotein (TRL) clearance via the LDL receptor-related protein^1^. Multiple studies suggest that purported interactions between *LPL* polymorphisms and BMI on triglycerides are consistent with quantile-dependent expressivity, in that greater adiposity is associated with higher average triglyceride concentrations. Fisher *et al*. first reported a significant interaction between *LPL* S291 and BMI on triglycerides (P_interaction_ = 0.02)^35^. Compared to those with a BMI < 25, their Fig. 2 showed heavier men had a greater triglyceride difference between genotypes (heavier vs. leaner: 0.42 vs. −0.17 mmol/L difference) corresponding to their higher average triglyceride (1.94 vs. 1.54 mmol/L) in the Northwich Park Heart Study II project^35^. The European Atherosclerosis Research Studies reported that S291-carriers had greater increases in plasma triglycerides with increasing BMI than non-carriers (P < 0.01)^36^. Correspondingly, the genotype differences and average triglyceride concentrations were −0.08 and 0.89 mmol/L in the lowest BMI tertile, respectively, 0.18 and 1.00 mmol/L in the intermediate BMI tertile, respectively, and 0.18 and 1.13 mmol/L in the highest BMI tertile, respectively. Mailly *et al*. reported a marginally greater triglyceride difference for carriers vs. non-carriers of the N9 mutation in overweight men (0.53 ± 0.27 mmol/L difference) with higher average triglycerides (1.86 ± 0.05 mmol/L) than in leaner men (0.02 ± 0.26 mmol/L difference) with lower average triglycerides (1.51 ± 0.05 mmol/L)^37^, as did Gerdes *et al*. for the highest BMI tertile (0.25 mmol/L genotype difference) with higher average triglycerides (1.12 mmol/L) vis-à-vis leaner men (0.10 mmol/L genotype difference) with lower average triglycerides (0.93 mmol/L)^36^, although neither reached statistical significance.

**Figure 6:**
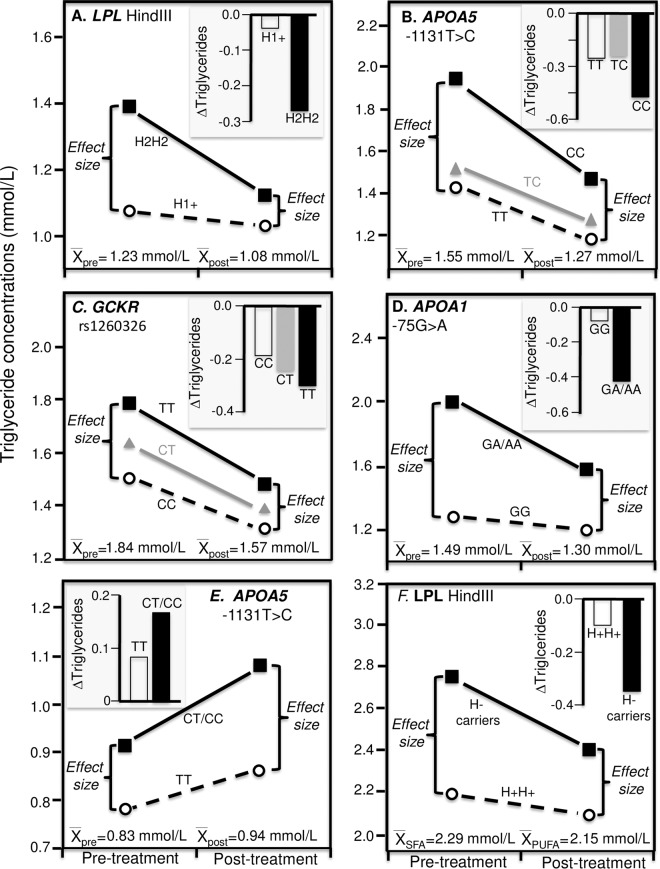
Precision medicine perspective of different mean changes in triglyceride concentrations by genotypes (histogram inserts) vs. quantile-dependent expressivity perspective of larger genetic effect size when average triglycerides concentrations were high vs. low requiring nonparallel changes in triglycerides by genotype, for: (**A**) Jenaa *et al*.’s 1997 report on the triglyceride response to 10-week weight loss diet in 58 H2H2 homozygotes and 57 H1-carriers of the of the *LPL* Hind III polymorphism (P = 0.03)^38^, (**B**) Yamasaki *et al*.’s 2015 report on the effect of a 3 month weight loss intervention in 87 TT, 163 TC, and 43 CC genotypes of the *APOA5* -1131T > CT polymorphism^39^, (**C**) Pollin *et al*.’s 2011 reported on the effect of a one-year lifestyle intervention in 919 subjects by the *GCKR* rs1260326 P446L polymorphism^44^; (**D**) Ruaño *et al*.’s report on the effect of 6-month exercise training in 53 homozygotes and 22 A-allele carriers of the *APOA1* −75 G/A polymorphism^57^; (**E**) Lin *et al*.’s report on the effect of going from a 54% carbohydrate/31% fat diet to a 70% carbohydrate/15% fat diet on 36 TT and 20 C-carriers of the *APOA5* -1131T > C polymorphism^68^; (**F**) Humphries *et al*.’s 1996 report on the triglyceride response to a high saturated fat (26% SFA, 10% MUFA, 2% PUFA) and high polyunsaturated fat diets (9% SFA, 6% MUFA, 23% PUFA) in 45 H + and 10 H- genotypes of the *LPL Hin*dlll gene loci^69^.

Figure 6A presents Jemaa *et al*.’s findings for a 10 week restricted calorie diet by the *LPL* HindIII polymorphism^38^. From a precision medicine perspective, the histogram (insert) shows plasma triglyceride concentration decreased significantly more in H2H2 homozygotes than H1-carriers (0.27 vs. 0.04 mmol/L decreases, P = 0.03). Consistent with quantile-dependent expressivity, the difference between genotypes was greater at baseline than after weight loss (0.32 ± 0.13 vs. 0.09 ± 0.11 mmol/L) in accordance with the higher average triglycerides at baseline (1.23 ± 0.07 vs. 1.08 ± 0.05 mmol/L). Again, the smaller genetic effect size at the lower (post-treatment) than higher (pre-treatment) triglyceride concentrations require that the effects of the genotypes do not move in parallel when triglycerides are decreased by weight loss. Subtracting the pre-treatment from the post-treatment triglyceride levels will necessarily create a relatively greater triglyceride decrease for the genotype with the higher pre-treatment triglyceride level vis-à-vis the genotype with the lower pre-treatment level.

Figure 6B presents Yamasaki *et al*.’s results for a 3-month lifestyle weight loss intervention that reduced dietary calorie intake from 2066 ± 27 to 1691 ± 22 kcal/d, increased energy expenditure from 2100 ± 34 to 2266 ± 36 kcal/d, and reduced BMI from 25.65 ± 0.18 to 24.75 ± 0.17 kg/m^2^^39^. The intervention produced twice as much triglyceride decrease in CC than CT or TT genotypes of the *APOA5* -1135T > C polymorphism. Quantile-dependent expressivity would attribute the difference to the smaller genotype differences after weight loss than before (CC vs. TT difference ± SE: 0.19 ± 0.12 after vs. 0.43 ± 0.19 mmol/L before) due to the lower average triglyceride concentrations after weight loss (1.27 ± 0.04 vs. 1.55 ± 0.05 mmol/L).

Data extracted from Vohl *et al*.’s Fig. 1 showed that triglycerides increased significantly with increasing adiposity in *LPL* HindIII H + homozygotes but not H- carriers as measured by BMI (slope ± SE: 0.137 ± 0.052 vs. 0.020 ± 0.041 mmol/L per kg/m^2^), and visceral adipose tissue area (0.009 ± 0.003 vs. 0.001 ± 0.004 mmol/L per cm^2^)^40^. The difference between genotypes increased with increasing BMI (0.12 ± 0.07 mmol/L per kg/m^2^, P = 0.08), and visceral area (0.008 ± 0.005 mmol/L per cm^2^, P = 0.10), and average triglycerides concentrations also increased with increasing BMI (0.069 ± 0.033 mmol/L per kg/m^2^, P = 0.04) and visceral area (0.006 ± 0.003 mmol/L per cm^2^, P = 0.02). In another study, Senti *et al*.’s data showed that women with higher waist-to-hip ratios had somewhat greater triglyceride difference between H- carriers and H+ homozygotes (0.255 mmol/L difference) than women with lower waist-to-hip ratios (0.194 mmol/L difference), which probably corresponds to their difference in average triglycerides concentrations (1.26 vs. 0.90 mmol/L, respectively, P < 0.001)^41^.

Huang *et al*. reported that the triglyceride difference between SS and SX/XX genotypes of the *LPL* S447X polymorphism was greater in centrally obese than nonobese twins (0.24 vs. 0.06 mmol/L differences, P = 0.16), corresponding to the greater average triglycerides in centrally obese than nonobese twins (1.39 vs. 0.99 mmol/L)^42^. Garenc *et al*.’s data showed that the triglyceride difference between homozygotes for the S477-allele and X477-carriers was greater in obese men (0.80 mmol/L difference, P = 0.002) and women (0.53 mmol/L difference, P = 0.01) than normal weight men (0.09 mmol/L difference) and women (0.15 mmol/L difference), which corresponds with the higher average triglycerides of the obese men (2.02 mmol/L) and women (1.25 mmol/L) compared to the normal weight men (1.15 mmol/L) and women (1.10 mmol/L)^43^. Cole *et al*.’s reported significantly greater effect size for *LPL* rs12678919 in obese than lean subjects (β = −0.148 ± 0.03 vs. −0.050 ± 0.03 mmol/L per G allele, P_interaction_ = 0.0007) whose average triglycerides differed by > 0.5 mmol/L^26^.

The *GCKR* Pro446Leu polymorphism (rs1260326) affects triglyceride concentrations by increasing hepatic glucokinase activity^44^. Pollin *et al*. reported that there was a significant interaction (P = 0.04) between the triglyceride response to lifestyle intervention and rs1260326, with the 446L (T)-allele showing enhanced triglyceride reduction (P = 0.04, Fig. 6C)^44^. Consistent with quantile-dependent expressivity, triglycerides increased more per T-allele at baseline when triglycerides averaged 1.84 mmol/L (β = 0.141 mmol/L, P = 1.8 × 10^−9^) than after weight loss intervention when estimated triglycerides averaged 1.57 mmol/L (β = 0.084 mmol/L). This agrees with Cole *et al*.’s report of a significantly greater effect size in obese than lean subjects (β = 0.093 ± 0.03 vs. 0.067 ± 0.02 mmol/L per T allele, P_interaction_ = 0.03) whose average triglycerides differed by > 0.5 mmol/L^26^.

Clausen *et al*. reported that the *IRS-1* G972R mutation and obesity interacted to significantly increase plasma triglyceride concentrations (P_interaction_ = 0.04)^45^. The difference between the R-carriers and GG homozygotes was seven-fold greater in obese than lean subjects (0.70 ± 0.42 vs. 0.10 ± 0.09 mmol/L), which could be due to the higher average triglyceride concentrations of the obese subjects (1.46 ± 0.10 vs. 0.91 ± 0.02 mmol/L). Baroni *et al*. also reported that R-carriers showed a greater difference from GG homozygotes in obese subjects (2.11–1.73 = 0.38 mmol/L difference) than lean subjects (1.72–1.78 = −0.06 mmol/L difference) corresponding to the higher average triglyceride concentrations of the obese vs. lean subjects (1.80 ± 0.10 vs. 1.73 ± 0.09 mmol/L)^46^.

Zhi *et al*. reported that differences between the CC, CT, and TT genotypes of the *MTHFR* C677T polymorphism were significantly greater (P = 0.02) in women with BMI > 24 kg/m^2^ (0.90, 0.99, 1.09 mmol/L, respectively) who had higher average triglycerides (1.00 mmol/L), than in leaner women (0.65, 0.71, 0.63 mmol/L, respectively) who had low triglycerides (0.67 mmol/L)^47^. Our analyses of Yin *et al*.’s data (their Fig. 3) showed significant differences between obese and lean Chinese for the effects of the *MTHFR* C677T genotype (0.387 vs. 0.029 mmol/L per dose of the T allele, P_interaction_ = 0.006) and *PCSK9* E670G (AG-AA difference: 1.64 vs. −0.17 mmol/L, P_interaction_ < 0.0001) consistent with the higher average triglycerides of the overweight/obese Chinese (1.74 vs. vs. 1.21 mmol/L)^48^.

Other gene-environment interactions involving body weight also appear attributable to quantile-dependent effects. With respect to the *APOB* XbaI polymorphism, Turner *et al*.’s data showed the effect size per dose of the X+ allele became progressively greater when going from the lowest (−0.012 mmol/L), to the intermediate (0.035 mmol/L) to highest BMI tertile (0.053 mmol/L, P_interaction_ = 0.015)^49^. The lowest BMI tertiles had low average triglycerides (estimated as 0.84 mmol/L), the intermediate BMI tertiles had intermediate average triglycerides (0.89 mmol/L) and the highest BMI tertile had the highest average triglycerides (1.01 mmol/L).

Jemaa *et al*. reported that triglyceride concentrations were significantly lower for *APOE* ε3ε3 homozygotes than ε2- or ε4-carriers only in Tunisians who had BMI ≥ 30 kg/m^2^ (and presumably higher triglycerides)^50^.

Becer *et al*. reported that triglyceride concentrations were more strongly related to the dose of the A-alleles of the *PPARγ*2 Pro12Ala polymorphism in obese (0.115 mmol/L per A allele, P = 0.05) than nonobese subjects (0.054 mmol/L per A allele, P = 0.52) which probably relates to the higher average triglyceride concentrations of the obese subjects (1.71 ± 0.03 vs. 1.16 ± 0.03 mmol/L)^51^.

Finally, Stojkovic *et al*. reported a significantly stronger trend (P = 0.01) from the GG, CG, to CC genotypes of the *PNPLA3* rs738409 polymorphism in overweight (i.e., 1.39, 1.50, 1.57 mmol/L for BMI > 25) than normal weight subjects (from 1.26, 1.20, 1.20 mmol/L for BMI ≤ 25, P_interaction_ = 0.003) consistent with the higher triglycerides in the overweight than normal weight subjects (1.54 vs. 1.20 mmol/L)^52^.

### Physical activity

Aerobic physical activity decreases triglyceride concentrations by facilitating triglyceride hydrolysis and use by skeletal muscles^88^. Meta-analyses suggest that triglyceride concentrations average 0.11 mmol/L less for those who walked ≥6000 vs. <2000 steps/day, and 0.23 mmol/L less for those who exercised at 50% of VO_2_max for three 30-minute sessions per week compared to less active subjects^89^. Our analyses of Senti *et al*.’s data^53^ showed that the each dose of the H+ allele of the *LPL* HindIII polymorphism was associated with a triglyceride increase of 0.148 mmol/L in the least active men (expending ≤291 kcal/d), 0.135 mmol/L in men expending 292–525 kcal/d, and 0.105 mmol/L in the most active men (>525 kcal/d) in an apparent gene-environment interaction. However, average triglyceride concentrations decreased with increasing physical activity: from 1.432, 1.250, to 1.106 mmol/L, respectively, suggesting an effect size for the H+ allele consistent with quantile-dependent expressivity.

Pisciotta *et al*. reported that the −265 T/C polymorphism of the *APOA*2 gene had a greater effect on triglyceride concentrations in sedentary men (TT, TC, CC: 2.12, 1.64, 1.24 mmol/L) than active men who cycled 120–150 km/wk (1.35, 1.33, 1.09 mmol/L, respectively), consistent with the higher triglyceride-cholesterol concentrations of the sedentary men (1.74 ± 0.11 vs. 1.30 ± 0.08 mmol/L)^54^.

Tanisawa *et al*. reported that triglyceride concentrations increased with increasing tertiles of their GRS_TG_ (1st: 0.93 ± 0.06, 2^nd^: 1.41 ± 0.13, 3^rd^: 1.46 ± 0.14 mmol/L) in Japanese men with low cardiorespiratory fitness, but not in those with higher fitness (1st: 0.92 ± 0.07, 2^nd^: 0.77 ± 0.06, 3^rd^: 1.05 ± 0.08 mmol/L, P_interaction_ = 0.03) as predicted by quantile-dependent expressivity given the higher average triglycerides of the low vs. high fitness groups (1.30 ± 0.07 vs. 0.93 ± 0.04 mmol/L)^55^.

A small training study by Hagberg *et al*. reported larger triglyceride decreases in the +/− and +/+ than −/− genotypes of the *LPL* Pvull polymorphism (−0.68 ± 0.28 vs. −0.35 ± 0.18 mmol/L)^56^. However, training reduced average triglyceride concentrations from 2.04 to 1.41 mmol/L, and quantile-dependent expressivity would therefore predict the larger genotype difference at baseline than follow-up (+/− and +/+ vs. −/− difference: 0.25 vs. −0.08 mmol/L, respectively), producing nonparallel triglyceride decreases by genotype.

Ruaño *et al*. reported that 6 months of supervised aerobic exercise training produced significantly greater percent reductions in triglyceride concentration in A-carriers of the *APOA1* -75G > A polymorphism than in GG homozygotes (P = 0.05)^57^. Figure 6D shows that average triglyceride concentrations were lower after training than before (1.30 vs. 1.49 mmol/L) corresponding to smaller genotypic differences after training than before (0.38 vs. 0.72 mmol/L).

### Smoking

Smokers are insulin resistant and exhibit impaired lipid metabolism, including impaired triglyceride clearance after a mixed meal^90^. Meta-analyses suggest that triglyceride concentrations of smokers average 9.1% higher than nonsmokers, and show a dose-dependent relationship from light (10.7%), moderate (11.5%) to heavy smokers (18%)^91^. Quantile-dependent expressivity would predict greater genetic effects on triglycerides in smokers than nonsmokers because of the smokers’ higher triglyceride concentrations. Czerwinski *et al*. in fact reported that the heritability of plasma triglyceride concentrations was higher in smokers (*h*^2^ = 0.70, average triglycerides 1.68 ± 0.06) than nonsmokers (*h*^2^ = 0.42, average triglycerides 1.58 ± 0.03)^58^. With respect to individual loci, smoking is reported to modify the effects on triglycerides of the upstream stimulatory factor 1 (*USF1*) gene polymorphism rs2516839^59^, C242T polymorphism of the cytochrome b-245 alpha chain (*CYBA*) gene^60^, –482 C > T in the insulin-responsive element of *APOC3*^61^, *LPL* HindIII^53,62^, and *LPL* rs263^63^.

There are several reports of *LPL* polymorphisms affecting the triglyceride response to smoking. Peacock *et al*. found larger differences between H+H+ homozygotes and H- carriers of the *LPL*– HindIII polymorphism in smokers than nonsmokers (sexes combined: 0.23 vs. 0.01 mmol/L difference, P < 0.02) consistent with the smokers’ higher average triglyceride concentrations (1.03 vs. 0.92 mmol/L)^62^. Senti *et al*. reported a significant difference between H+ H+ homozygotes and H- carriers of the *LPL* HindIII polymorphism in sedentary smokers (0.53 ± 0.26 mmol/L, P = 0.04) but not nonsmokers (0.05 ± 0.14 mmol/L), again, consistent with the higher average triglycerides of the sedentary smokers (1.54 ± 0.12 vs. 1.39 ± 0.07 mmol/L)^53^. Pyun *et al*.’s data showed a greater difference between CC homozygotes and T-carriers for *LPL* rs271 in smokers (2.20–1.96 = 0.24 mmol/L) than nonsmokers (1.66–1.63 = 0.03 mmol/L, P_interaction_ = 0.009) consistent with the smokers’ higher average triglycerides (2.11 vs. 1.65 mmol/L)^63^.

Niemiec *et al*. reported that the *USF1* rs2516839 polymorphism modified the triglyceride response to smoking, however, triglyceride differences between the CC, CT and TT genotypes were greater in smokers (2.27 ± 0.26, 1.80 ± 0.09, 1.53 ± 0.10 mmol/L, respectively) in accordance with their higher average triglycerides (1.79 ± 0.07 mmol/L) than in nonsmokers (1.49 ± 0.11, 1.46 ± 0.06, 1.57 ± 0.08, respectively) in accordance with their lower concentrations (1.51 ± 0.05 mmol/L)^59^. Ge *et al*. reported that the difference between the CC homozygotes and carriers of the T-allele of *CYBA* C242T polymorphism was significant in smokers (0.17 mmol/L, P = 0.01) but not nonsmokers (0.04 mmol/L, P = 0.76), which quantile-dependent expressivity would partially attribute to the smokers higher average triglyceride concentrations (1.33 vs. 1.21 mmol/L)^60^. Waterworth *et al*. reported that the smoking-triglyceride relationship was modified by both *APOC3* –482 C > T (P_interaction_ = 0.009) and 3238 C > G polymorphisms (P_interaction_ = 0.04)^61^. Specifically, smokers’ had higher average triglyceride concentrations than nonsmokers (1.74 vs. 1.59 mmol/L), and as predicted, a greater effect per dose of the –482T-allele (0.135 vs. −0.009 mmol/L) and per dose of the 3238G-allele (0.380 vs. 0.113 mmol/L, calculated from their table two) than nonsmokers^61^.

Smokers did not have higher triglycerides than nonsmokers in the 41,000 subjects of the Population Architecture Using Genomics and Epidemiology (PAGE) study (mean ± SE: 1.476 ± 0.010 vs. 1.486 ± 0.005 mmol/L)^92^. Consistent with quantile-dependent expressivity, their meta-analysis did not show any significant SNP by smoking interactions.

### Diet

Each 1% isoenergetic replacement of carbohydrates with fat is expected to decrease plasma triglyceride concentrations by an average of 0.021 mmol/L if saturated, 0.019 if monounsaturated, and 0.026 mmol/L if polyunsaturated^93^. Adherence to a Mediterranean diet decreases plasma triglyceride concentrations by an average of 0.069 mmol/L^94^. Quantile-dependent expressivity would predict larger genetic effects on low-fat high-carbohydrate diets than high-fat low-carbohydrate diets, and larger genetic effects on Western than Mediterranean diets, in accordance with the expected higher triglycerides of the former.

Gomez-Delgado *et al*. reported that decreases in plasma triglyceride due to adopting a Mediterranean diet were significantly greater in 203 GG homozygotes of the tumor necrosis factor alpha gene (*TNFA*, rs1800629) than in 48 carriers of the A-allele, i.e. approximately 0.31 vs. 0.12 mmol/L, respectively (P = 0.005)^64^. However, plasma triglyceride concentrations averaged approximately 1.80 mmol/L at baseline and 1.52 mmol/L on the diet, and correspondingly, the differences between the GG and GA/AA genotypes were 0.38 vs. 0.19 mmol/L, respectively. A quantile-dependent interpretation of these results is that the Mediterranean diet decreased plasma triglyceride concentrations, which in turn produced a smaller difference between genotypes.

Pyun *et al*.’s data showed a greater triglyceride difference between CC homozygotes and T-carriers for *LPL* rs263 with increasing energy intake: 0.005 mmol/L difference for ≤1500 kcal/d, 0.14 mmol/L difference for 1501–2000 kcal/d, 0.13 mmol/L for 2001–2500 kcal/d, and 0.20 mmol/L for > 2500 kcal/d, P_interaction_ = 0.02) corresponding to the increasing average triglycerides concentrations with energy intake (1.73, 1.78, 1.78, 1.84 mmol/L, respectively)^63^.

Garcia-Rios *et al*. reported significant interactions between plasma concentrations of n-6 polyunsaturated fatty acids and *LPL* rs238 (P_interaction_ = 0.05) and *LPL* rs1059611 (P_interaction_ = 0.04)^65^. Below median n-6 PUFA concentrations, the rs1059611 triglyceride difference between AA homozygotes and carriers of the G allele was 0.33 mmol/L and the average triglyceride concentration across genotypes was 2.14 mmol/L. Above the median, the genotype difference was smaller (−0.09 mmol/L) in accordance with lower average triglyceride concentrations (1.37 mmol/L), consistent with quantile-dependent expressivity. Nearly identical results were reported for rs238, which was in strong linkage disequilibrium with rs1059611.

Garcia-Rios *et al*. also reported a significant interaction between plasma saturated fatty acids concentrations and the circadian clock gene Period 2 (*PER2*) rs2304672 on plasma triglyceride concentrations (P_interaction_ = 0.004)^66^. Above the median plasma SFA concentration of 30.9 mmol/L, plasma triglyceride concentrations differed significantly between the carriers of the G allele and CC homozygotes (2.61–1.98 =  0.63 mmol/L, P = 0.001) but not below the median (1.43–1.51 = −0.08 mmol/L), consistent with the higher average triglyceride concentrations in those with the high plasma SFA concentrations (2.06 ± 0.06 vs. 1.50 ± 0.06 mmol/L).

Samoan triglyceride concentrations were elevated (average 1.18 mmol/L) if they consumed a modern dietary pattern, intermediate for a transitional dietary pattern (average 0.96 mmol/L), and low for a neo-traditional diet (average 0.83 mmol/L)^67^. Correspondingly, the difference between CC homozygotes and carriers of the T allele of the insulin induced gene 2 (*INSIG2*) rs9308762 differed significantly on the modern diet (0.88 mmol/L), showed intermediate difference for transitional diet (0.33 mmol/L), and showed no significant difference (−0.10 mmol/L) on the neo-traditional diet (P_interaction_  = 0.04)^67^.

Figure 6E presents Lin *et al*.’s report of a two-fold greater triglyceride increase in C-carriers of the *APOA5* -1131T > C polymorphism vs. TT homozygotes in going from a 54% carbohydrate/31% fat diet to a 70% carbohydrate/15% fat diet^68^. Consistent with quantile-dependent expressivity, the genotype difference went from 0.13 ± 0.10 to 0.22 ± 0.10 mmol/L while average triglycerides increased from 0.83 ± 0.08 to 0.94 ± 0.05 mmol/L.

Figure 6F displays the significantly greater triglyceride decreases in *LPL Hin*dlll H- carriers than H+ homozygotes when switching from a high saturated fat to a high polyunsaturated fat diet (0.35 vs. 0.10 mmol/L decreases, P = 0.05) reported by Humphries *et al*.^69^ However, the high polyunsaturated fat diet produced smaller differences between H- and H+ genotypes than the high saturated fat diet (0.31 vs. 0.56 mmol/L) in accordance with its lower average triglyceride concentrations (2.15 vs. 2.29 mmol/L).

**Figure 7:**
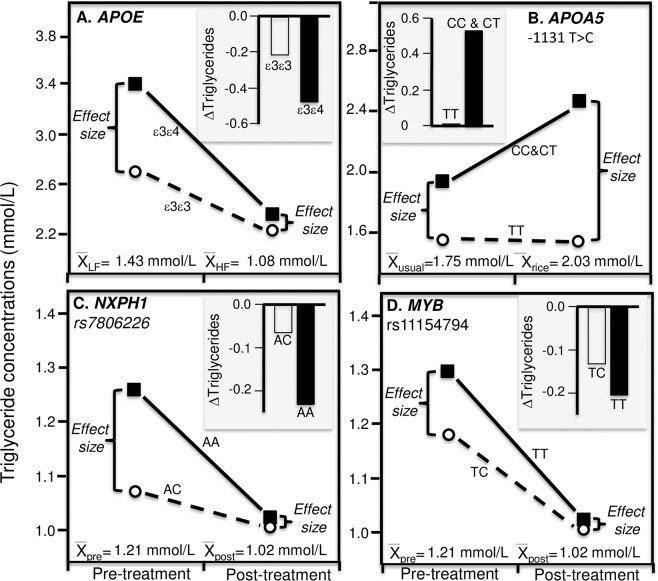
Precision medicine perspective of different mean changes in triglyceride concentrations by genotypes (histogram inserts) vs. quantile-dependent expressivity perspective of larger genetic effect size when average triglycerides concentrations were high vs. low requiring nonparallel changes in triglycerides by genotype, for: (**A**) Carvalho-Wells *et al*.’s 2012 report on the triglyceride response to switching from a low-fat (24% fat, 59% carbohydrate) to high-fat diet (38% fat, 45% carbohydrate with 3.45 g DHA/d) in 44 *APOE* ε3ε3 homozygotes vs. 44 ε3ε4 heterozygotes (P_interaction_ = 0.03)^70^; (**B**) Kang *et al*.’s 2014 report of switching from their usual to a refined rice diet in 43 TT homozygotes and 50 C carriers of the *APOA5* -1131 T > C polymorphism^71^. (**C**) Vallée Marcotte *et al*.’s 2016 report on starting omega-3 (n-3) fatty acid supplementation in 142 AA homozygotes and 66 AC heterozygotes of the neurexophilin-1 (*NXPH1*) rs7806226 polymorphism^72^; (**D**) Vallée Marcotte *et al*.’s 2016 report on starting omega-3 (n-3) fatty acid supplementation in 155 TT homozygotes and 53 CT heterozygotes of the V-MYB avian myeloblastosis viral oncogene homolog (MYB) rs11154794^72^.

Figure 7A presents Carvalho-Wells *et al*.’s finding that switching from a low-fat diet to a high-fat diet containing 3.45 g/d DHA produced significantly greater triglyceride reductions in *APOE* ε3ε4 heterozygotes (−0.48 ± 0.11 mmol/L) than ε3ε3 homozygotes (−0.22 ± 0.06 mmol/L, P_interaction_ = 0.03). Average triglyceride concentrations were higher on the low-fat than high-fat diet (1.43 vs. 1.08 mmol/L), and the difference between genotypes was correspondingly greater on the low-fat than the high fat diet (0.33 vs. 0.06 mmol/l difference)^70^.

Figure 7B presents Kang *et al*.’s report of significantly greater triglyceride increases from a refined rice diet in carriers of C-allele than TT homozygotes of the *APOA5* -1131 T > C polymorphism (0.53 vs. −0.01 mmol/L, P = 0.02)^71^. Again, the difference between genotypes was greater after the diet than before (0.92 ± 0.04 vs. 0.38 ± 0.03 mmol/L difference) when average triglycerides were higher (2.03 ± 0.02 vs. 1.75 ± 0.01 mmol/L).

Finally, Fig. 7C,D present Vallée Marcotte *et al*.’s report of a significantly different triglyceride responses to 5 g/day of fish oil by the neurexophilin-1 (*NXPH1*) rs7806226 polymorphism (P_interaction_ = 0.004) and V-MYB avian myeloblastosis viral oncogene homolog (*MYB*) rs11154794 polymorphism (P = 0.02)^72^. The histograms present the greater triglyceide reductions in homozygotes. The difference between genotypes was greater at baseline when average triglycerides concentrations were higher (1.21 ± 0.04 mmol/L) than after treatment when average concentrations were lower (1.02 ± 0.04 mmol/L) for both rs7806226 (AA-AC: 0.187 ± 0.07 vs. 0.018 ± 0.08 mmol/L) and rs11154794 (TT-TC: 0.087 ± 0.111 vs. 0.014 ± 0.075 mmol/L), consistent with quantile-dependent expressivity.

### Alcohol

Triglycerides increase an average of 0.11 mmol/L per 23 g/d of alcohol consumed, equivalent to 1 large beer^89^. Although De Vries *et al*. study of 394,584 subjects revealed no gene-alcohol interactions attaining genomewide significance^73^, there are several reports of larger genetic effects in drinkers than nondrinkers in accordance with their higher average triglyceride concentrations and quantile-dependent expressivity. These include those by Ruixing *et al*. for *APOC3* 3238 C > G (rs5128) genotypes in drinkers (CC/CG/GG: 0.97, 0.95, 1.28 mmol/L, P < 0.001) vs. nondrinkers (CC/CG/GG: 0.91, 1.01, 0.93 mmol/L, P = 0.002)^74^, by Yin *et al*. for GG vs. A-carriers of the *APOA5* 457 G > A (rs3135507) polymorphism in drinkers (1.01 vs. 0.95) vs. nondrinkers (0.97 vs. 0.99 mmol/L, P_Interaction_ < 0.001)^75^, by Pyun *et al*.’s between *LPL* rs263 GG homozygotes and A-carriers in drinkers (1.97–1.78 = 0.19 mmol/L) vs. nondrinkers (1.65–1.64 = 0.01 mmol/L, P_interaction_ = 0.009)^63^, and by Zhou *et al*. for the cholesteryl ester transfer protein (*CETP*) TaqIB polymorphism (rs708272) in drinkers (B1B1/B1B2/B2B2: 1.42, 1.01, 0.88 mmol/L, P = 0.02) than nondrinkers (0.94, 1.17, 0.99 mmol/L, respectively, P = 0.18)^76^. In each case, average triglyceride concentrations were greater in drinkers than nondrinker (Ruixing and Yin *et al*.: 1.09 ± 0.03 vs. 0.97 ± 0.03 mmol/L, P = 0.01^74,75^; Pyun *et al*.: estimated 1.90 vs. 1.65 mmol/L^63^; Zhou *et al*.: 1.17 ± 0.09 vs. 1.04 ± 0.06 mmol/L^76^).

Tan *et al*. deduced a significant interaction between alcohol intake and the aldehyde dehydrogenase 2 gene (*ALDH2)* in their effect on triglyceride concentrations (P = 3.3 × 10^−5^)^77^. Specifically, the triglyceride difference between GG homozygotes and A carriers increased from nondrinkers (−0.09 mmol/L), to drinkers consuming 1–10 g/d (0.15 mmol/L), 10–30 g/d (0.26 mmol/L), to ≥30 g/d (0.51 mmol/L). However, average triglyceride concentrations also increased from nondrinkers (1.21 mmol/L), 1–10 g/d (1.21 mmol/L), 10–30 g/d (1.42 mmol/L), to ≥30 g/d (1.48 mmol/L) in support of a quantile-dependent expressivity.

### Insulin resistance

VLDL overproduction due to diminished degradation of newly synthesized apo B, increased free fatty acid flux to the liver, and increased de novo hepatic lipogenesis all contribute to hypertriglyceridemia in T2DM^86^. Klimentidis *et al*.^28^ reported that the effect of GRS_TG_ on triglyceride concentrations increased progressively with increasing tertiles of fasting insulin (estimated β = 0.15, β  = 0.21, β  = 0.23, P = 2.7 × 10^−11^) and HOMA-IR (estimated β = 0.14, β = 0.21, β  = 0.24, P = 2.5 × 10^−11^), in the context of a highly significant triglyceride increases with both (fasting insulin: P = 2.4 × 10^−100^; HOMA-IR: P = 9.1 × 10^−133^). Justesen *et al*. also reported that higher HOMA-IR was associated with a greater affect of GRS_TG_ on triglycerides concentration (P_interaction_ = 0.0009), presumably in association with rising average triglyceride concentrations, although the triglyceride-HOMA-IR relationship was not reported^30^.

Inamdar *et al*. reported that T2DM patients, who had higher average triglycerides than non-T2DM patients (1.90 vs. 1.27 mmol/L), showed greater carrier-noncarrier triglyceride differences for *APOE* ε2 (0.56 vs. 0.31) and ε4 (−0.45 vs. −0.12 mmol/L)^78^. Data presented by Vohl *et al*. showed that the fasting triglyceride difference between *LPL*-HindIII H + H + homozygotes and H-carriers were greater for fasting insulin concentrations ≥71.5 than  < 71.5 pmol/L (0.65 vs. −0.12 mmol/L), consistent with the higher triglyceride concentrations of the former (2.01 vs. 1.18 mmol/L)^40^.

### Pregnancy

There is a two-fold increase in circulating triglyceride levels during the third trimester due to enhanced VLDL-production and LPL supression^86^. Ma *et al*. reported that the effect of LPL deficiency had a much greater effect during pregnancy, when triglycerides are normally two- to three-fold higher, than when not pregnant, i.e., the LPL deficient women’s triglyceride were 20.2–22.5 mmol/L when pregnant vs. 3.4 mmol/L when not^79^.

### Twin studies

Higher average triglyceride concentrations in MZ vs. DZ twins (1.33 vs. 1.07 mmol/L) could have contributed to the higher triglyceride correlations (r_MZ_ = 0.527 vs. r_DZ_ = 0.349) reported by Jermendy *et al*., affecting their estimation of genetic and environmental influences^80^.

### Limitations

An important limitation of the analysis of the Framingham data its reliance on the simple formula *h*^*2*^ = 2β_OP_/(1 + r_spouse_) and *h*^*2*^ =  [(1 + 8r_spouse_β_FS_)^0.5^ − 1]/(2r_spouse)_ to estimate heritability^12^. These formula are unlikely to embody the true complexity of triglyceride inheritance. With respect to the published examples cited, we wish to emphasize that consistency with quantile-dependent expressivity does not disprove gene-environment interactions, rather, it provides an alternative interpretation. The examples presented are those originally interpreted from the perspective of precision medicine and biological interactions that might be more easily explained by quantile-dependent expressivity. It is not our contention that all triglyceride gene-environment interactions are explained by quantile-dependent expressivity. For example, Wojczynski *et al*.’s report of the significant effect (P < 0.0001) of the *APOB* rs676210 variant on the triglyceride response to fenofibrate would not be attributable to the quantile-dependent expressivity of Fig. 1 due to their being larger genotype differences post-treatment when triglycerides were low than pretreatment when triglycerides were high^95^. Some gene-environmental interactions may arise because triglycerides and environmental factors may be coregulated by shared genes or genes in strong linkage equilibrium. For that reason, the examples presented in Figs. 4–7 may be particularly informative for testing whether the genetic effect size is affected by average triglyceride concentrations because they represent intervention affecting triglyceride concentrations directly. Among the various genetic variants discovered to date, the proportion of the total triglyceride heritability explained by any specific SNP is too small to noticeably affect *h*^*2*^^5,6^. Thus quantile-dependence of triglyceride heritability estimated from parent and offspring phenotypes does not necessarily describe the interactions between any particular genetic variant and its environment. Many published reports do not provide the information required to evaluate their consistency with quantile-dependent expressivity, namely unadjusted triglyceride concentrations by genotype and condition.

In conclusion, assuming Falconer and Mackay’s formula apply^12^, these analyses suggest that triglyceride heritability is strongly dependent upon whether an individual is high or low relative to the triglyceride distribution in the population. Alternatively, quantile-dependent shared environmental effects could also give rise to the increase in β_OP_ and β_FS_ with increasing average triglyceride concentrations, however our previous findings showing increasing genetic effect size for GRS_TG_2 and during post-prandial triglyceride increases^3^, and the studies cited herein^13–79^ support a genetic interpretation. Quantile-dependent expressivity potentially provides a common principle underlying a plethora of published gene-drug and gene-environment interactions. Specifically, rather than attributing these interactions on the basis of triglyceride metabolism, gene functionality, and the specific metabolic effect of adiposity, physical activity, insulin resistance, diet, smoking, alcohol, and pregnancy, quantile-dependent expressivity postulates that the impaired functionalities of these genetic variants are simply triglyceride concentration dependent.

## Data Availability

The data used in these analyses are available data directly from the National Institutes of Health at https://biolincc.nhlbi.nih.gov/studies/framcohort/, https://biolincc.nhlbi.nih.gov/studies/gen3/ and https://biolincc.nhlbi.nih.gov/studies/framoffspring/ with requestor’s full or expedited IRB review.

## References

[1] Expert Panel on Detection (2001). Evaluation, and Treatment of High Blood Cholesterol in Adults Executive Summary of the Third Report of The National Cholesterol Education Program (NCEP) Expert Panel on Detection, Evaluation, and Treatment of High Blood Cholesterol in Adults (Adult Treatment Panel III). JAMA..

[2] Williams PT (2012). Quantile-specific penetrance of genes affecting lipoproteins, adiposity and height. PLoS One..

[3] Williams Paul T. (2020). Quantile-dependent expressivity of postprandial lipemia. PLOS ONE.

[4] Williams Paul T. (2020). Quantile-Specific Heritability may Account for Gene–Environment Interactions Involving Coffee Consumption. Behavior Genetics.

[5] Teslovich TM (2010). Biological, clinical and population relevance of 95 loci for blood lipids. Nature..

[6] Willer CJ (2013). Discovery and refinement of loci associated with lipid levels. Nat. Genet..

[7] Elder SJ (2009). Genetic and environmental influences on factors associated with cardiovascular disease and the metabolic syndrome. J. Lipid. Res..

[8] Koenker R, Hallock KF (2001). Quantile regression. J. Economic Perspectives..

[9] Gould WW (1992). Quantile regression with bootstrapped standard errors. Stata Technical Bulletin..

[10] Kannel WB, Feinleib M, McNamara PM, Garrison RJ, Castelli WP (2006). An investigation of coronary heart disease in families. The Framingham offspring study. Am. J. Epidemiol..

[11] Splansky GL (2007). The Third Generation Cohort of the National Heart, Lung, and Blood Institute’s Framingham Heart Study: design, recruitment, and initial examination. Am. J. Epidemiol..

[12] Falconer, D. S. & Mackay, T. F. C. Introduction to Quantative Genetics. 4th edition. 2004 Pearson Education Limited. London ISBN 978-81-317-2740-9. page 176

[13] Aslibekyan S (2012). Variants identified in a GWAS meta-analysis for blood lipids are associated with the lipid response to fenofibrate. PLoS One..

[14] Brautbar A (2011). Variants in the APOA5 gene region and the response to combination therapy with statins and fenofibric acid in a randomized clinical trial of individuals with mixed dyslipidemia. Atherosclerosis.

[15] Lai CQ (2007). Fenofibrate effect on triglyceride and postprandial response of apolipoprotein A5 variants: the GOLDN study. Arterioscler. Thromb. Vasc. Biol..

[16] Perez-Martinez P (2009). Association between glucokinase regulatory protein (GCKR) and apolipoprotein A5 (APOA5) gene polymorphisms and triacylglycerol concentrations in fasting, postprandial, and fenofibrate-treated states. Am. J. Clin. Nutr..

[17] Cardona F (2009). The -1131T > C SNP of the APOA5 gene modulates response to fenofibrate treatment in patients with the metabolic syndrome: a postprandial study. Atherosclerosis..

[18] Irvin MR (2010). Apolipoprotein E polymorphisms and postprandial triglyceridemia before and after fenofibrate treatment in the Genetics of Lipid Lowering Drugs and Diet Network (GOLDN) study. Circ. Cardiovasc. Genet..

[19] Brisson D (2002). Effect of apolipoprotein E, peroxisome proliferator-activated receptor alpha and lipoprotein lipase gene mutations on the ability of fenofibrate to improve lipid profiles and reach clinical guideline targets among hypertriglyceridemic patients. Pharmacogenetics..

[20] Pedro-Botet J (2001). Apolipoprotein E genotype affects plasma lipid response to atorvastatin in a gender specific manner. Atherosclerosis..

[21] Carmena R, Roederer G, Mailloux H, Lussier-Cacan S, Davignon J (1993). The response to lovastatin treatment in patients with heterozygous familial hypercholesterolemia is modulated by apolipoprotein E polymorphism. Metabolism..

[22] Anagnostopoulou K (2007). Pharmacogenetic study of cholesteryl ester transfer protein gene and simvastatin treatment in hypercholesterolaemic subjects. Expert Opin. Pharmacother..

[23] Tuteja S (2018). Genetic variants associated with plasma lipids are associated with the lipid response to niacin. J Am. Heart Assoc..

[24] Balakrishnan S (2002). Apolipoprotein E gene polymorphism alters lipids before pancreas transplantation. Transplantation..

[25] Cabello I (2018). Association of APOA5 and APOC3 genetic polymorphisms with severity of hypertriglyceridemia in patients with cutaneous T-cell lymphoma treated with bexarotene. JAMA. Dermatol..

[26] Cole CB (2014). Adiposity significantly modifies genetic risk for dyslipidemia. J. Lipid Res..

[27] Ali A (2016). Do genetic factors modify the relationship between obesity and hypertriglyceridemia? Findings from the GLACIER and the MDC studies. Circ. Cardiovasc. Genet..

[28] Klimentidis YC, Arora A (2016). Interaction of insulin resistance and related genetic variants with triglyceride-associated genetic variants. Circ. Cardiovasc. Genet..

[29] Zubair N (2014). Genetic risk score and adiposity interact to influence triglyceride levels in a cohort of Filipino women. Nutr. Diabetes..

[30] Justesen JM (2015). Interactions of lipid genetic risk scores with estimates of metabolic health in a Danish population. Circ. Cardiovasc. Genet..

[31] Ahmad S (2018). Adiposity and genetic factors in relation to triglycerides and triglyceride-rich lipoproteins in the Women’s Genome Health Study. Clin. Chem..

[32] Wu Y (2013). Genetic association with lipids in Filipinos: waist circumference modifies an APOA5 effect on triglyceride levels. J Lipid Res.

[33] Kim M, Yoo HJ, Lee HJ, Lee JH (2019). Longitudinal interaction between APOA5 -1131T > C and overweight in the acceleration of age-related increase in arterial stiffness through the regulation of circulating triglycerides. Hypertens. Res..

[34] Hsu MC (2013). Central obesity in males affected by a dyslipidemia-associated genetic polymorphism on APOA1/C3/A4/A5 gene cluster. Nutr. Diabetes..

[35] Fisher RM (1995). Interaction of the lipoprotein lipase asparagine 291– > serine mutation with body mass index determines elevated plasma triacylglycerol concentrations: a study in hyperlipidemic subjects, myocardial infarction survivors, and healthy adults. J. Lipid Res..

[36] Gerdes C (1997). Lipoprotein lipase variants D9N and N291S are associated with increased plasma triglyceride and lower high-density lipoprotein cholesterol concentrations: studies in the fasting and postprandial states: the European Atherosclerosis Research Studies. Circulation..

[37] Mailly F (1996). Association between the LPL-D9N mutation in the lipoprotein lipase gene and plasma lipid traits in myocardial infarction survivors from the ECTIM Study. Atherosclerosis..

[38] Jemaa R, Tuzet S, Betoulle D, Apfelbaum M, Fumeron F (1997). Hind III polymorphism of the lipoprotein lipase gene and plasma lipid response to low calorie diet. Int. J. Obes. Relat. Metab. Disord..

[39] Yamasaki M (2015). The interaction of Apolipoprotein A5 gene promoter region T-1131C polymorphism (rs12286037) and lifestyle modification on plasma triglyceride levels in Japanese. Nutr. Res. Pract..

[40] Vohl MC (1995). The lipoprotein lipase HindIII polymorphism modulates plasma triglyceride levels in visceral obesity. Arterioscler. Thromb. Vasc. Biol..

[41] Sentí M (2000). Relationship of abdominal adiposity and dyslipemic status in women with a common mutation in the lipoprotein lipase gene. The REGICOR investigators. Atherosclerosis..

[42] Huang AQ (2006). Lipoprotein lipase gene S447X polymorphism modulates the relation between central obesity and serum lipids, a twin study. Int. J. Obes. (Lond)..

[43] Garenc C (2000). Linkage and association studies of the lipoprotein lipase gene with postheparin plasma lipase activities, body fat, and plasma lipid and lipoprotein concentrations: the HERITAGE Family Study. Metabolism..

[44] Pollin TI (2011). Triglyceride response to an intensive lifestyle intervention is enhanced in carriers of the GCKR Pro446Leu polymorphism. J. Clin. Endocrinol. Metab..

[45] Clausen JO (1995). Insulin resistance: interactions between obesity and a common variant of insulin receptor substrate-1. Lancet..

[46] Baroni MG (2001). The G972R variant of the insulin receptor substrate-1 (IRS-1) gene, body fat distribution and insulin-resistance. Diabetologia..

[47] Zhi X (2016). Gender-specific interactions of MTHFR C677T and MTRR A66G polymorphisms with overweight/obesity on serum lipid levels in a Chinese Han population. Lipids Health. Dis..

[48] Yin RX (2012). Several genetic polymorphisms interact with overweight/obesity to influence serum lipid levels. Cardiovasc. Diabetol..

[49] Turner PR, Talmud PJ, Visvikis S, Ehnholm C, Tiret L (1995). DNA polymorphisms of the apoprotein B gene are associated with altered plasma lipoprotein concentrations but not with perceived risk of cardiovascular disease: European Atherosclerosis Research Study. Atherosclerosis..

[50] Jemaa R (2006). Apolipoprotein E polymorphism in the Tunisian population: frequency and effect on lipid parameters. Clin. Biochem..

[51] Becer E, Çırakoğlu A (2017). Effect of the Pro12Ala Polymorphism of the Peroxisome Proliferator-activated Receptor γ2 Gene on Lipid Profile and Adipokines Levels in Obese Subjects. Balkan J. Med. Genet..

[52] Stojkovic IA (2014). The PNPLA3 Ile148Met interacts with overweight and dietary intakes on fasting triglyceride levels. Genes Nutr..

[53] Sentí M (2001). Physical activity modulates the combined effect of a common variant of the lipoprotein lipase gene and smoking on serum triglyceride levels and high-density lipoprotein cholesterol in men. Hum. Genet..

[54] Pisciotta L (2003). Physical activity modulates effects of some genetic polymorphisms affecting cardiovascular risk in men aged over 40 years. Nutr Metab Cardiovasc Dis..

[55] Tanisawa K (2014). Polygenic risk for hypertriglyceridemia is attenuated in Japanese men with high fitness levels. Physiol. Genomics..

[56] Hagberg JM, Ferrell RE, Dengel DR, Wilund KR (1999). Exercise training- induced blood pressure and plasma lipid improvements in hypertensives may be genotype dependent. Hypertension.

[57] Ruaño G (2006). Apolipoprotein A1 genotype affects the change in high density lipoprotein cholesterol subfractions with exercise training. Atherosclerosis..

[58] Czerwinski SA (2004). Gene by smoking interaction: evidence for effects on low-density lipoprotein size and plasma levels of triglyceride and high-density lipoprotein cholesterol. Hum. Biol..

[59] Niemiec P (2015). The rs2516839 polymorphism of the USF1 gene may modulate serum triglyceride levels in response to cigarette smoking. Int. J. MolSci..

[60] Ge J, Ding Z, Song Y, Wang F (2012). Smoking dose modifies the association between C242T polymorphism and prevalence of metabolic syndrome in a Chinese population. PLoS One..

[61] Waterworth DM (2000). Contribution of apolipoprotein C-III gene variants to determination of triglyceride levels and interaction with smoking in middle-aged men. Arterioscler. Thromb. Vasc. Biol..

[62] Peacock RE, Temple A, Gudnason V, Rosseneu M, Humphries SE (1997). Variation at the lipoprotein lipase and apolipoprotein AI-CIII gene loci are associated with fasting lipid and lipoprotein traits in a population sample from Iceland: interaction between genotype, gender, and smoking status. Genet. Epidemiol..

[63] Pyun JA (2012). Interaction effects of lipoprotein lipase polymorphisms with lifestyle on lipid levels in a Korean population: A Cross-sectional Study. Genomics. Inform..

[64] Gomez-Delgado F (2014). Polymorphism at the TNF-alpha gene interacts with Mediterranean diet to influence triglyceride metabolism and inflammation status in metabolic syndrome patients: From the CORDIOPREV clinical trial. Mol. Nutr. Food. Res..

[65] Garcia-Rios A (2011). Genetic variations at the lipoprotein lipase gene influence plasma lipid concentrations and interact with plasma n-6 polyunsaturated fatty acids to modulate lipid metabolism. Atherosclerosis..

[66] Garcia-Rios A (2012). A Period 2 genetic variant interacts with plasma SFA to modify plasma lipid concentrations in adults with metabolic syndrome. J. Nutr..

[67] Baylin A (2013). INSIG2 variants, dietary patterns and metabolic risk in Samoa. Eur. J. Clin. Nutr..

[68] Lin J (2011). Elevated levels of triglyceride and triglyceride-rich lipoprotein triglyceride in- duced by a high-carbohydrate diet is associated with polymorphisms of APOA5-1131T.C and APOC3-482C.T in Chinese healthy young adults. Ann. Nutr. Metab..

[69] Humphries SE, Talmud PJ, Cox C, Sutherland W, Mann J (1996). Genetic factors affecting the consistency and magnitude of changes in plasma cholesterol in response to dietary challenge. QJM..

[70] Carvalho-Wells AL, Jackson KG, Lockyer S, Lovegrove JA, Minihane AM (2012). APOE genotype influences triglyceride and C-reactive protein responses to altered dietary fat intake in UK adults. Am. J. Clin. Nutr..

[71] Kang R, Kim M, Chae JS, Lee SH, Lee JH (2014). Consumption of whole grains and legumes modulates the genetic effect of the APOA5-1131C variant on changes in triglyceride and apolipoprotein A-V concentrations in patients with impaired fasting glucose or newly diagnosed type 2 diabetes. Trials..

[72] Vallée Marcotte B (2016). Novel genetic loci associated with the plasma triglyceride response to an omega-3 fatty acid supplementation. J. Nutrigenet. Nutrigenomics..

[73] de Vries PS (2019). Multi-ancestry genome-wide association study of lipid levels incorporating gene-alcohol interactions. Am. J. Epidemiol..

[74] Ruixing Y (2010). Interactions of the apolipoprotein C-III 3238C > G polymorphism and alcohol consumption on serum triglyceride levels. Lipids Health Dis..

[75] Yin RX, Li YY, Liu WY, Zhang L, Wu JZ (2011). Interactions of the apolipoprotein A5 gene polymorphisms and alcohol consumption on serum lipid levels. PLoS One..

[76] Zhou Y, Yin R, Deng Y, Li Y, Wu J (2008). Interactions between alcohol intake and the polymorphism of rs708272 on serum high-density lipoprotein cholesterol levels in the Guangxi Hei Yi Zhuang population. Alcohol..

[77] Tan A (2012). A genome-wide association and gene–environment interaction study for serum triglycerides levels in a healthy Chinese male population. Hum. Mol. Genet..

[78] Inamdar PA, Kelkar SM, Devasagayam TP, Bapat MM (2000). Apolipoprotein E polymorphismin non-insulin-dependent diabetics of Mumbai, India and its effect on plasmalipids and lipoproteins. Diabetes. Res. Clin. Pract..

[79] Ma Y (1993). Gene-environment interaction in the conversion of a mild-to-severe phenotype in a patient homozygous for a Ser172– > Cys mutation in the lipoprotein lipase gene. J. Clin. Invest..

[80] Jermendy G (2011). Effect of genetic and environmental influences on cardiometabolic risk factors: a twin study. Cardiovasc Diabetol..

[81] Lipid Research Clinics Program. Manual of laboratory operations. Vol 1. Lipid and lipoprotein analysis. Washington, DC: US Government Printing Office. (DHEW publi- cation no. (NIH) 75-628), (1974).

[82] Karlin S, Cameron EC, Williams PT (1981). Sibling and parent-offspring correlation estimation with variable family size. Proc. Natl. Acad. Sci. USA.

[83] Hegele RE, Pollex RL (2007). Apolipoprotein A-V genetic variation and plasma lipoprotein response to fibrates. Arterioscler. Thromb. Vasc. Biol..

[84] Scarisbrick JJ (2013). U.K. consensus statement on safe clinical prescribing of bexarotene for patients with cutaneous T-cell lymphoma. Br. J. Dermatol..

[85] Dattilo AM, Kris-Etherton PM (1992). Effects of weight reduction on blood lipids and lipoproteins: a meta-analysis. Am. J. Clin. Nutr..

[86] Miller M (2011). Triglycerides and cardiovascular disease: a scientific statement from the American Heart Association. Circulation..

[87] Guardiola M, Ribalta J (2017). Update on APOA5 genetics: toward a better understanding of its physiological impact. Curr. Atheroscler. Rep..

[88] Miller M (2011). Triglycerides and cardiovascular disease: a scientific statement from the American Heart Association. Circulation..

[89] Hata Y, Nakajima K (2000). Life-style and serum lipids and lipoproteins. J. Atheroscler. Thromb..

[90] Eliasson B, Mero N, Taskinen M, Smith U (1997). The insulin resistance syndrome and postprandial lipid intolerance in smokers. Atherosclerosis..

[91] Craig WY, Palomaki GE, Haddow JE (1989). Cigarette smoking and serum lipid and lipoprotein concentrations: an analysis of published data. BMJ..

[92] Dumitrescu L (2013). No evidence of interaction between known lipid-associated genetic variants and smoking in the multi-ethnic PAGE population. Hum. Genet..

[93] Mensink RP, Zock PL, Kester AD, Katan MB (2003). Effects of dietary fatty acids and carbohydrates on the ratio of serum total to HDL cholesterol and on serum lipids and apolipoproteins: a meta-analysis of 60 controlled trials. Am. J. Clin. Nutr..

[94] Kastorini CM (2011). The effect of Mediterranean diet on metabolic syndrome and its components: a meta-analysis of 50 studies and 534,906 individuals. J. Am. Coll. Cardiol..

[95] Wojczynski MK (2010). Apolipoprotein B genetic variants modify the response to fenofibrate: a GOLDN study. J. Lipid. Res..

